# Radiomics-Based Computed Tomography Urogram Approach for the Prediction of Survival and Recurrence in Upper Urinary Tract Urothelial Carcinoma

**DOI:** 10.3390/cancers16183119

**Published:** 2024-09-10

**Authors:** Abdulsalam Alqahtani, Sourav Bhattacharjee, Abdulrahman Almopti, Chunhui Li, Ghulam Nabi

**Affiliations:** 1 School of Medicine, Centre for Medical Engineering and Technology, University of Dundee, Dundee DD1 9SY, UK; axalqahtani@dundee.ac.uk (A.A.); 140022587@dundee.ac.uk (A.A.); 2 Radiology Department, College of Applied Medical Sciences, Najran University, Najran 55461, Saudi Arabia; 3 School of Veterinary Medicine, University College Dublin, D04 W6F6 Dublin, Ireland; sourav.bhattacharjee@ucd.ie; 4 School of Science and Engineering, University of Dundee, Dundee DD1 4HN, UK; c.li@dundee.ac.uk

**Keywords:** radiomics, CT urogram, UTUC, texture analysis, recurrence, survival, prognosis

## Abstract

**Simple Summary:**

In this study, a computed tomography (CT)-based radiomics model was developed to improve predictions of survival and recurrence in patients with upper tract urothelial carcinoma (UTUC)—a rare and aggressive cancer. Notably, an accurate prediction of survival and recurrence is essential for deciding on effective therapeutic management scheme(s) in UTUC. The combined radiomics features extracted from CT scans with clinical data significantly improved the accuracy of predicting patient outcomes compared to clinical data alone. The combined clinico-radiomics model also demonstrated a superior predictive performance to the models developed using only clinical or radiomics data, achieving high concordance indices for survival and recurrence prediction. The integrated approach unraveled hidden patterns in CT data indicative of the recurrence risk and enabled precise patient risk stratification to inform treatment planning. It also provided a deeper understanding of the tumor matrix and its evolution, enabling more refined, tailored, and (potentially) more effective treatment plans for UTUC patients.

**Abstract:**

Upper tract urothelial carcinoma (UTUC) is a rare and aggressive malignancy with a poor prognosis. The accurate prediction of survival and recurrence in UTUC is crucial for effective risk stratification and guiding therapeutic decisions. Models combining radiomics and clinicopathological data features derived from computed tomographic urograms (CTUs) can be a way to predict survival and recurrence in UTUC. Thus, preoperative CTUs and clinical data were analyzed from 106 UTUC patients who underwent radical nephroureterectomy. Radiomics features were extracted from segmented tumors, and the Least Absolute Shrinkage and Selection Operator (LASSO) method was used to select the most relevant features. Multivariable Cox models combining radiomics features and clinical factors were developed to predict the survival and recurrence. Harrell’s concordance index (C-index) was applied to evaluate the performance and survival distribution analyses were assessed by a Kaplan–Meier analysis. The significant outcome predictors were identified by multivariable Cox models. The combined model achieved a superior predictive accuracy (C-index: 0.73) and higher recurrence prediction (C-index: 0.84). The Kaplan–Meier analysis showed significant differences in the survival (*p* < 0.0001) and recurrence (*p* < 0.002) probabilities for the combined datasets. The CTU-based radiomics models effectively predicted survival and recurrence in the UTUC patients, and enhanced the prognostic performance by combining radiomics features with clinical factors.

## 1. Introduction

Upper tract urothelial carcinoma (UTUC) is a rare and aggressive malignancy originating in the lining of the renal pelvis and ureter, accounting for approximately 5–10% of all urothelial carcinomas [1]. Despite its relatively low incidence, UTUC presents significant challenges in oncological practice, characterized by high local recurrence rates, intravesical relapse, and distant metastasis [2,3]. Collectively, these factors contribute to a poor prognosis, underscoring the critical need for improved prognosis [4]. Computed tomography urograms (CTUs), the primary imaging modality, offer high diagnostic accuracy but fall short in preoperative staging [5,6,7]. Additionally, magnetic resonance urograms (MRUs) show promise in tumor grading and staging, although they are typically reserved for cases where CTUs are contraindicated [1,8,9]. Ureteroscopic biopsy and urine cytology provide insights into tumor characteristics with limited information on recurrence risk [10,11,12,13].

UTUC prognosis varies depending on the treatment approach and disease attributes. Non-surgical intervention, often chosen due to patient factors, results in poor outcomes, with a median survival of two years [14,15]. Radical nephroureterectomy (RNU), the gold standard treatment, yields better outcomes with five-year cancer-specific survival (CSS) rates of 74–87% [16,17,18]. The prognosis after RNU depends on both preoperative (hydronephrosis, smoking history, genetic markers) [1,19,20,21,22,23] and postoperative factors (tumor stage and grade, tumor size) [24,25,26,27]. The five-year CSS rates vary from 94% for Ta tumors to 13% for T4 and 88% for low-grade to 57% for high-grade tumors [24,25]. This limitation underscores the need for improved prognostic models to guide personalized treatment strategies in UTUC.

Radiomics, an emerging field at the intersection of medical imaging and data science, offers a promising solution to these challenges. By extracting and analyzing large numbers of quantitative features from medical images, it has the potential to uncover subtle patterns and tumor characteristics that may elude conventional visual interpretation [28,29,30]. It presents a promising avenue for improving the comprehension of tumor biology and behavior by capturing intricate characteristics of tumor texture, heterogeneity, and morphology [31]. The application of radiomics analysis has gained widespread adoption across various medical fields, including oncology [32,33,34], cardiovascular [35,36], and neurology diseases [37,38], with diverse imaging platforms, including CT, MRI, single-photon emission computed tomography (SPECT), and positron emission tomography (PET) [39].

However, its utility in smaller tumors such as UTUCs, often ≤2 cm in size and with limited datasets, needs further enhancement. Despite some exploratory radiomics approaches demonstrating efficacy in discriminating tumor grade and stage in UTUC [40], the application of radiomics to predict survival and recurrence in UTUC remains largely unexplored and leaves a gap in the literature. The application of radiomics presents a unique opportunity to improve UTUC prognostication. By leveraging advanced feature extraction techniques and machine learning algorithms, it harbors the potential to develop robust predictive models that can capture the complex biology of UTUC tumors.

This study aimed to develop CT-based radiomics models for improved prognostication in UTUC. The current work sought to create predictive models combining radiomics features with clinicopathological information to assess their accuracy in predicting both 5-year death and recurrence risks. By comparing these models against traditional prognostic tools, this study aimed to enhance clinical decision-making and facilitate personalized treatment strategies, potentially improving UTUC management and patient outcomes.

## 2. Materials and Methods

### 2.1. Study Design and Data

This retrospective study adhered to the Standards for Reporting Diagnostic Accuracy Studies (STARD) guidelines and the CheckList for EvaluAtion of Radiomics (CLEAR) for radiomics studies. CTUs and clinicopathological data of UTUC patients who underwent RNU from January 2000 to December 2022 were accessed from the Tayside Urological Cancers (TUCAN) database under due approval from the East of Scotland Research Ethical Service (Approval No. IGTCAL-2023-075). Using the Caldicott Approval, the requirement for informed consent was waived.

**Figure 1:**
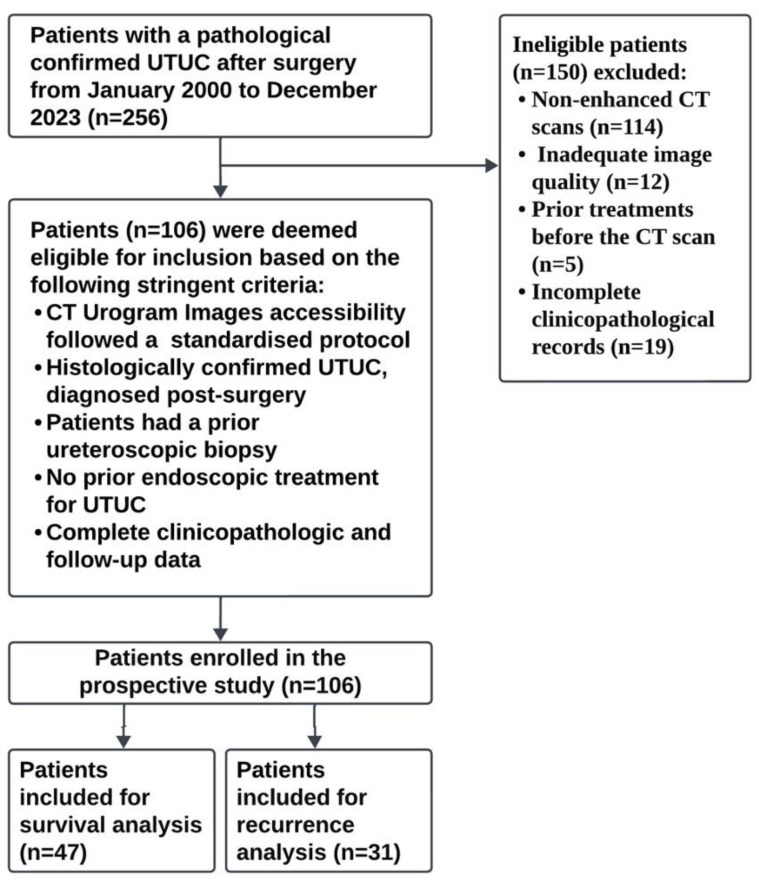
Workflow showing the flow of patient selection strategy followed in this study.

Of the 256 patient records in the TUCAN database, 106 met the inclusion criteria, which included access to the CTU dataset, adhering to a set protocol, histologically validated UTUC, and the absence of prior endoscopic management for UTUC before the CT assessment. A cluster of 150 patients were excluded from the study due to non-contrast CT scans (114 cases), poor-quality images (12 cases), treatments before their CT scans (5 cases), and missing clinical/pathology data (19 cases). The primary outcomes were comprehensive survival predictions for 46 cases and recurrence predictions for 31 cases (Supplementary Material S1). The pathological assessments were conducted by an experienced uropathologist and discussed during tumor board reviews (Figure 1).

This study included Ta-stage UTUC patients who underwent RNU despite conservative management being typically recommended for this stage. This decision was based on the presence of high-risk features associated with increased progression and recurrence risks. Inclusion criteria were multifocal tumors, tumor size > 2 cm, high-grade pathology, and hydronephrosis, which aligned with European Association of Urology (EAU) guidelines and was suggestive of more aggressive treatment for lower-stage cancers with such high-risk characteristics [41]. The decision for RNU in these cases was made with multidisciplinary tumor board approval, ensuring adherence to best clinical practices and guideline recommendations. The rationale behind this approach is detailed in the study protocol, reflecting a consensus among oncologists, radiologists, and pathologists involved in this research, ensuring that the chosen therapeutic approach was tailored to the individual patient’s risk profile and corroborated contemporary clinical practice guidelines.

### 2.2. CT Protocol

Images from the group were obtained using a GE Healthcare Helical CT scanner. The scan parameters included a large body Scan Field of View (SFOV), 0.7 s gantry rotation time, 1.25 mm slice thickness, 1375:1 pitch, 40 mm detector coverage, Noise Index of 30, and CT Dose Index Volume (CTDIvol) of 9.59 mGy. The X-ray tube voltage was set to 120 kVp, and the X-ray tube current was automatically adjusted based on the patient’s size. The scan protocol includes a non-contrast-prone KUB. A volume of 90 mL of contrast was injected over 15 min for contrast-enhanced images. Supine abdomen/pelvis pictures were taken over 100 s, with 60 mL of contrast injected at a rate of 3 mL/s.

### 2.3. Follow-Up

Postoperative follow-up involved regular monitoring at specific intervals for the UTUC patients. Patients underwent evaluations every 3–4 months in the first year after nephroureterectomy. From the second to the third year, follow-up assessments were conducted at 6-month intervals. Beyond the third year, annual follow-ups were performed annually. The follow-up protocol comprised cystoscopy examination, routine blood tests, urinary cytology analyses, and chest and abdomen radiographic imaging. The two primary endpoints assessed were overall survival (OS), defined as the duration from the date of surgery until death from any cause, and recurrence rate, measured as the time elapsed between surgery and the occurrence of disease recurrence following nephroureterectomy.

### 2.4. Three-Dimensional Slicer for Tumor Segmentation

In this study, a systematic approach was adopted to manage DICOM images, executing 3D segmentation using the “grow from seeds” functionality in the 3D Slicer application (version 5.2.2) and then translating the segmented DICOM data into the NIfTI format, a common standard in neuroimaging informatics. Segmentation was conducted by a proficient radiologist (Reader 1) alongside a skilled urosurgical oncologist (Reader 2). The Dice Similarity Coefficient (DSC), with a threshold of 0.8, was employed to guarantee segmentation accuracy with at least 80% overlap accuracy. Only segmentations that met or exceeded this threshold were considered for further analysis. When segmentation did not meet this standard, the two readers collaborated to enhance the accuracy, while unresolved cases were excluded. For comparison, post-surgical histopathological evaluations served as the gold standard.

### 2.5. Radiomics Feature Extraction and Selection

**Figure 2:**
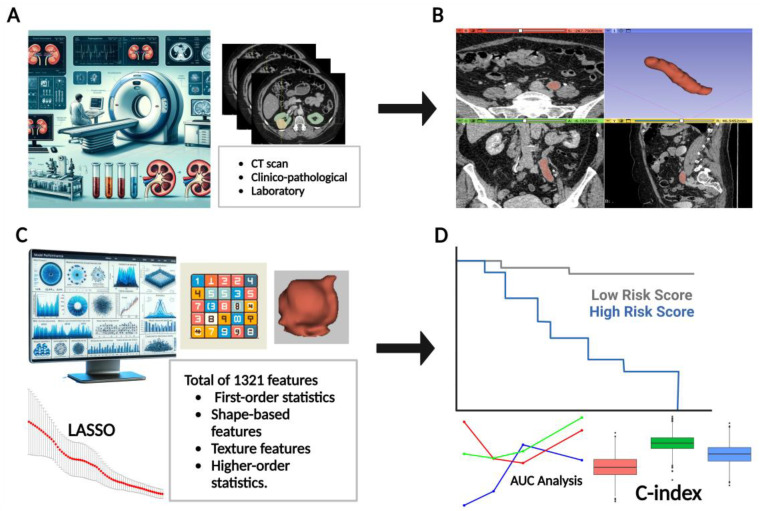
The radiomics workflow of this study. (**A**) High-quality CTU data were subjected to pre–processing; (**B**) a 3D ROI was segmented to include the entire tumor; (**C**) radiomics features were extracted from the segmented ROIs and subjected to feature selection; (**D**) multivariable prognostic models were developed by combining the selected features with clinical variables, and model performance was evaluated.

A comprehensive repertoire of quantitative features was extracted from the segmented regions of interest (ROIs) using the PyRadiomics library, an open-source Python package (version 3.7.9) dedicated to radiomics analysis. A total of 1324 features were computed, including first-order statistics, texture-based features from various matrices, and shape-based features. The class imbalance was addressed by the Synthetic Minority Over-sampling technique (SMOTE) algorithm, and features with an intraclass correlation coefficient (ICC) below 0.75 were excluded. Feature selection was performed using the Least Absolute Shrinkage and Selection Operator (LASSO) method to identify the most influential radiomics features. Image pre-processing included resampling to a voxel size of 1 × 1 × 1 mm^3^ and discretization with a fixed bin width of 25 Hounsfield Units. Original, filtered, and wavelet-transformed images were used for feature extraction (Figure 2).

### 2.6. Data Preparation and Modeling

This study initiated detailed data preparation—a crucial step that involved singling out clinical and non-numeric variables and exempting them from normalization. Z-score normalization was then applied to the numeric variables to standardize the data. A pivotal component was the correlation analysis to eliminate highly interdependent variables (with a correlation greater than 0.9) to ensure statistical independence. The dataset was bisected into clinical and radiomics variables for a focused analysis. An exhaustive dataset overview was prepared, detailing variables in both the clinical and radiomics categories.

Initially, features generated from the radiologist and urosurgical oncologist segmentations were evaluated for consistency using inter-rater reliability checks. Those with an ICC below 0.75 were excluded, narrowing the field to 163 features for further scrutiny. The Least Absolute Shrinkage and Selection Operator (LASSO) method was utilized to select the most influential features. LASSO is a regression analysis that enhances the model’s prediction accuracy and interpretability while effectively shrinking the less significant feature coefficients to zero.

This process retained only the most important predictors, simplifying the model and helping prevent overfitting. The retained features, deemed impactful by LASSO, clarified the feature selection process and its influence on predictive accuracy. A sub-analysis addressed concerns, including recurrence and carcinoma in situ (CIS), in multivariable models that may introduce bias due to their strong influence on survival. This analysis evaluated the interaction between radiomics features and clinical predictors when recurrence or CIS occurred.

We developed a Cox proportional hazards model using LASSO-selected variables, including all clinical factors (including recurrence and CIS) and selected radiomics features. This sub-analysis was performed on the entire cohort of 106 patients. Model performance was assessed using the concordance index (C-index), and the likelihood ratio test was used to evaluate overall model fit. The results of this sub-analysis were compared with those of the main analysis to assess the robustness of radiomics features in the presence of strong clinical predictors. The study workflow is summarized in Figure 2.

### 2.7. Evaluation and Statistical Analysis

This study employed a comprehensive statistical approach to investigate survival and recurrence outcomes across clinical, radiomics, and combined datasets. We began with Cox regression analysis, conducting univariate and multivariate analyses to assess individual and collective impacts of variables on survival and recurrence. LASSO Cox regression was then utilized to identify significant predictors and construct final Cox models for each dataset category and outcome type. We tested the proportional hazards assumption to ensure model validity and estimated survival and recurrence-free probabilities at specific time points.

Model performance was rigorously evaluated using multiple metrics, including the concordance index (C-index), Akaike Information Criterion (AIC), Bayesian Information Criterion (BIC), and Area Under the Curve (AUC) values at various time points for both survival and recurrence predictions. The analysis was further facilitated with extensive KM curve analysis, stratifying patients into high- and low-risk groups and assessing survival and recurrence-free probabilities over time. Bootstrapping techniques to generate C-index distributions and calculation of Brier scores for both outcome types were used to gauge model stability and accuracy.

This approach allowed the estimation of confidence intervals and assessed the consistency of model performance across different samples for survival and recurrence predictions. Furthermore, we conducted a comparative analysis of the models using AUC calculations and ROC curve visualizations at different durations, providing insights into their discriminative power over time for both outcomes. All analyses were performed using R statistical software (version 3.3.3), with a significance threshold set at *p* < 0.05.

## 3. Results

### 3.1. Patient Characteristics

The average age within the cohort (n = 106) was 74 years (49–93 years). Regarding the gender distribution, 44 patients (41% of participants) were female and 62 (58%) were male. For the smoking status, 25 patients (23%) never smoked, 32 (30%) were current smokers, and 49 (46%) were past smokers. BMI classifications: 35 subjects (33%) were in the normal range, 36 (34%) were overweight (pre-obesity), and 35 (33%) were obese. The cytology findings revealed 22 positives (20%), 31 negatives (29%), 10 questionable/doubtful (9%), and 43 undiagnosed (40%) cases. A total of 58 patients (54%) died, and 12 (11%) had metastases. All subjects (100%) obtained favorable results from CTUs. Left laterality was recorded in 43 (40%) and right laterality in 63 patients (59%). The condition was located in the renal pelvis in 60 (56%) and the ureter in 66 patients (62%). The histology grade ranged from poor (29%) to high (70%). The tumor stage distribution was T1 in 59 (55%), T2 in 19 (17%), and T3 or T4 in 28 (26%) patients. Carcinoma in situ (CIS) was recognized in 25 patients (23%). The survival rate was recorded in 47 cases (44%), whereas the recurrence rate was noted in 31 cases (29%; Table 1).

### 3.2. Development of Prognostic Models

The univariate and multivariate Cox regression analyses across clinical, radiomics, and combined datasets identified prognostic determinants in UTUC. The LASSO Cox regression and bootstrap resampling further refined these findings, confirming the stability and robustness of the identified predictors. A multivariate Cox proportional hazards model was used to assess the impact of clinical and radiomics features on survival and recurrence in UTUC, with the model fit confirmed using Schoenfeld residuals (Supplementary Material S1).

### 3.3. Advanced Clinico-Radiomics Prognostic Modeling for UTUC Survival 

This study employed a comprehensive statistical approach to elucidate prognostic factors for survival in UTUC patients, integrating both clinical and radiomics data. The multivariate Cox regression analysis of the clinical model identified several statistically significant predictors: tumor size (HR = 2.11, 95% CI: 2.95–62.19, *p* = 0.02), cytology (HR = 0.67, 95% CI: 0.46–0.98, *p* = 0.04), BMI (HR = 0.42, 95% CI: 0.19–0.94, *p* = 0.03), and multifocality (HR = 0.38, 95% CI: 0.16–0.90, *p* = 0.02). These findings highlighted the importance of traditional clinical factors in prognosticating UTUC outcomes.

In the radiomics model, wavelet.LHL_glcm_Correlation emerged as a marginally significant predictor (HR = 2.09, 95% CI: 2.59–98.60, *p* = 0.06), suggesting its potential relevance in survival prediction and underscoring the value of texture-based imaging features in capturing tumor characteristics. The combined clinico-radiomics model demonstrated enhanced predictive power, integrating clinical and imaging-derived features. This model identified the smoking status as a significant predictor with an apparent protective effect (HR = 0.67, 95% CI: 0.47–0.96, *p* = 0.03), a finding that warrants further investigation into the complex relationship between smoking and UTUC outcomes.

Additionally, two radiomics features were significantly associated with increased mortality: wavelet.LLH_glcm_InverseVariance (HR = 1.75, 95% CI: 1.08–2.84, *p* = 0.02) and wavelet.LHL_glcm_Correlation (HR = 1.78, 95% CI: 1.32–2.41, *p* = 0.0001). These texture-based features may capture underlying tumor heterogeneity and aggressiveness not apparent in conventional clinical assessments. Interestingly, hydronephrosis did not exhibit a statistically significant association with survival in the combined model (HR = 0.93, 95% CI: 0.44–1.97, *p* = 0.85), suggesting that its prognostic value may be diminished when considered alongside granular radiomics features (Table 2; Supplementary Material S1).

### 3.4. Assessment of Combined Clinico-Radiomics Model for UTUC Survival Prediction

**Figure 3:**
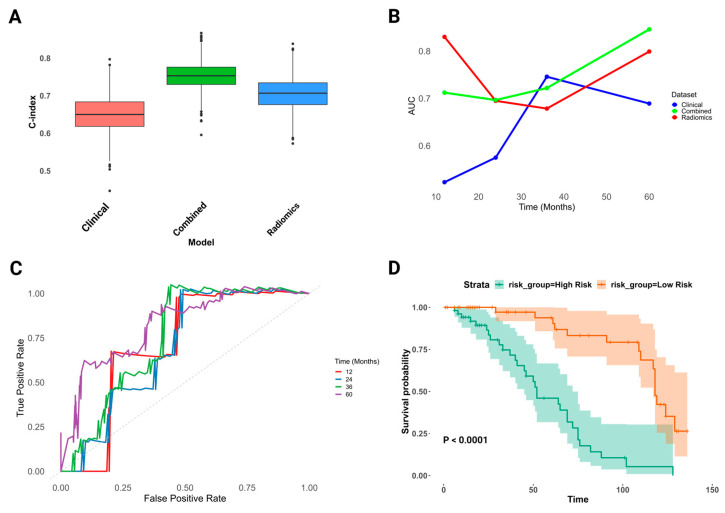
Evaluation of Cox proportional hazards regression models for UTUC survival prediction across clinical, radiomics, and combined datasets. (**A**) Distribution of C-index across bootstrap samples, demonstrating the enhanced predictive accuracy of the combined model compared to the clinical and radiomics models; (**B**) AUC over time for the combined, clinical, and radiomics models, highlighting the superior long-term prognostic performance of the combined model; (**C**) combined model ROC curves at various time points, showing high and stable predictive accuracy; (**D**) KM survival analysis of risk groups stratified by the combined model demonstrated significant (*p* < 0.05) differences in survival probabilities and reduced variability, due to superior discriminatory power.

The predictive model accuracies were evaluated through a 10-fold cross-validation. A LASSO Cox regression was employed for feature selection to ensure model stability and mitigate overfitting, confirming the importance of previously identified features and highlighting additional radiomics features in the combined dataset. The C-index, a measure of the predictive accuracy, was highest for the combined model (0.731, SE = 0.037), followed by the radiomics model (0.724, SE = 0.041), and the clinical model (0.638, SE = 0.047). The results were further validated through bootstrap resampling with 1000 iterations, yielding mean C-indices of 0.6496 for the clinical model, 0.7060 for the radiomics model, and 0.7520 for the combined model (Figure 3A).

The AIC and BIC were used to assess the model fit and complexity. The combined model demonstrated the best fit with the lowest AIC (304.26) and BIC (311.57), followed by the radiomics model (AIC: 304.98, BIC: 308.64), while the clinical model showed higher values (AIC: 318.24, BIC: 323.72). Proportional hazard assumptions were tested using Schoenfeld residuals. The global test for the combined model (*p* = 0.4366) indicated no significant violation of the proportional hazards assumption, supporting the model’s validity (Supplementary Material S1).

The predictive performance of Cox regression models was evaluated at various time intervals (12, 24, 36, and 60 months). The clinical model provided immediate predictive utility, diminishing over time, while the radiomics model demonstrated variable mid-to-long-term prognostic value. The combined model exhibited consistent, precise prognostication across timeframes (Figure 3B). In the AUC-ROC analysis, the clinical model showed moderate performance, with the AUCs ranging from 0.523 at 12 months to 0.6892 at 60 months. The radiomics model demonstrated superior short-term prediction with an AUC of 0.8301 at 12 months, maintaining a good performance up to 60 months (AUC: 0.7991).

The combined model showed consistently high performance across all time points, with AUCs ranging from 0.6966 at 24 months to 0.846 at 60 months, highlighting its robust predictive capability over time (Figure 3C). The KM analysis demonstrated statistically significant survival probability differences favoring the combined model (*p* < 0.0001) with reduced variability, highlighting its superior discriminative power (Figure 3D; Supplementary Material S1).

The interactions of key variables such as smoking status (HR = 0.6431, 95% CI: 0.4408–0.9383, *p* = 0.022) and wavelet.LLH_glcm_InverseVariance (HR = 2.5144, 95% CI: 1.1806–5.3553, *p* = 0.0168) were statistically significant (*p* < 0.05) due to independent contributions to the survival prediction. In contrast, the interaction terms, such as those between smoking status and wavelet.LHL_glcm_Correlation (HR = 1.0862, 95% CI: 0.7853–1.5024, *p* = 0.6173) and between hydronephrosis and wavelet.LLH_glcm_InverseVariance (HR = 0.5279, 95% CI: 0.2050–1.3593, *p* = 0.1855) were not statistically significant, suggesting that the combined effects of these clinical and radiomics variables were not multiplicative (Supplementary Materials Figures S3–S5).

### 3.5. Sub-Analysis: Interaction between Radiomics Features and Clinical Predictors

This study compared two prognostic models for UTUC: a main analysis model excluding recurrence and CIS, and a sub-analysis model including these factors. Several variables were significantly associated with survival outcomes in the main analysis, which excluded recurrence and CIS. The smoking status exerted a protective effect, with an HR of 0.67 (95% CI: 0.47–0.96, *p* = 0.03), suggesting a 33% reduction in the hazard of death for smokers. No significant association was found between hydronephrosis and survival (HR = 0.93, 95% CI: 0.44–1.97, *p* = 0.85).

**Figure 4:**
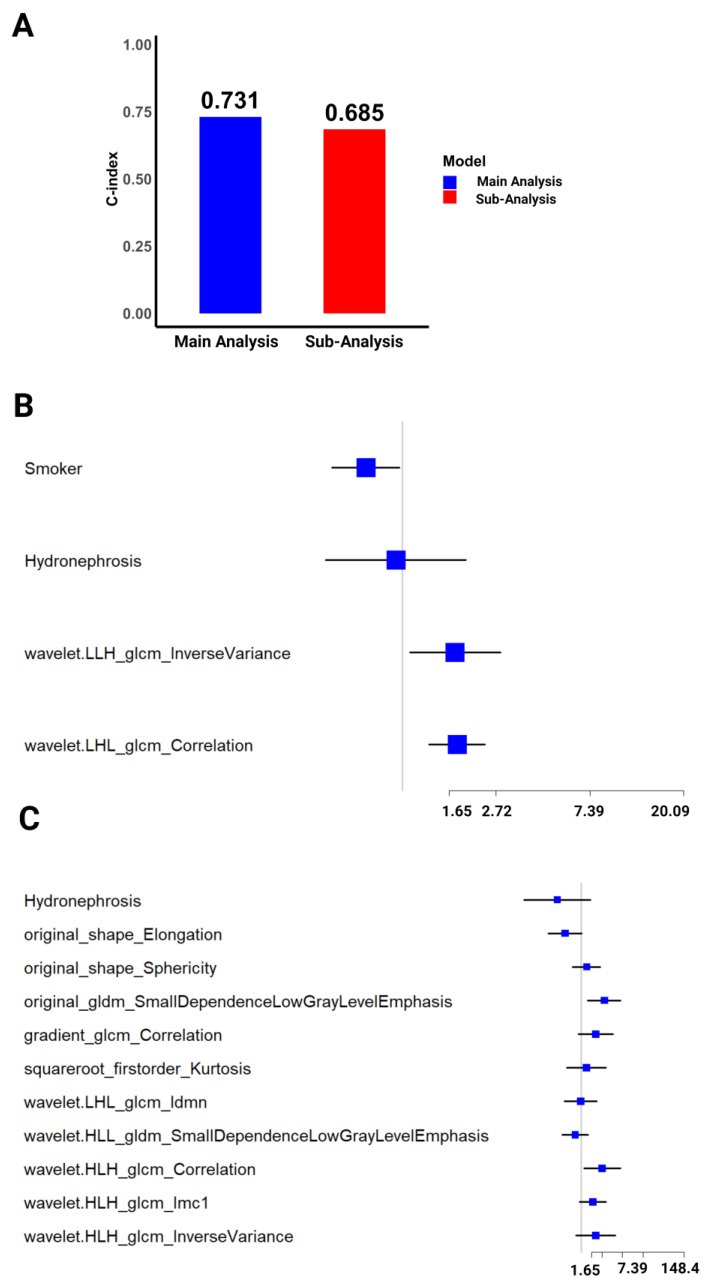
Visual comparison of the results from the main and sub-analysis of the combined model. (**A**) The C-indices of the main and sub-analyses of the combined dataset. Forest plots showing the HRs (with 95% CIs) of various predictors obtained after (**B**) main and (**C**) sub-analysis of the combined dataset. The blue squares represent the HR estimates (±95% CI), while the vertical line (HR = 1) implies no effect.

Two radiomics features demonstrated significant associations with an increased risk of death: wavelet.LLH_glcm_InverseVariance (HR = 1.75, 95% CI: 1.08–2.84, *p* = 0.023) and wavelet.LHL_glcm_Correlation (HR = 1.79, 95% CI: 1.32–2.41, *p* = 0.0001). The model exhibited good discriminatory power with a C-index of 0.731 and a highly significant likelihood ratio test (*p* < 0.0001). In the sub-analysis, which included recurrence and CIS, different patterns of association were observed. Hydronephrosis showed a non-significant protective effect (HR = 0.31, 95% CI: 0.06–1.55, *p* = 0.15). The radiomics feature original_shape_Elongation demonstrated a trend towards significance (HR = 0.45, 95% CI: 0.2–1.03, *p* = 0.06), potentially indicating a protective effect. Significant associations with increased hazard were found for original_gldm_SmallDependenceLowGrayLevelEmphasis (HR = 3.05, 95% CI: 1.37–6.77, *p* = 0.006) and wavelet.HLH_glcm_Correlation (HR = 2.75, 95% CI: 1.136–6.7, *p* = 0.02; Table 3). The main analysis of the combined model demonstrated superior performance with a C-index of 0.731. In contrast, the sub-analysis model showed slightly reduced discrimination (C-index: 0.685) and marginal statistical significance (likelihood ratio test, *p* = 0.06). This comparison highlights the complex interplay between clinical factors and radiomics features in UTUC prognostication (Figure 4A).

Forest plots were utilized to visually represent the hazard ratios and their 95% CIs for both analyses. In the main analysis, the radiomics features wavelet.LLH_glcm_InverseVariance and wavelet.LHL_glcm_Correlation were highlighted as being significantly associated with increased hazard (Figure 4B). The sub-analysis forest plot emphasized the significance of original_gldm_SmallDependenceLowGrayLevelEmphasis and wavelet.HLH_glcm_Correlation in the context of specific clinical characteristics, including recurrence and CIS (Figure 4C).

### 3.6. Advanced Clinico-Radiomics Prognostic Modeling for UTUC Recurrence

A comprehensive statistical analysis was conducted to evaluate the predictive value of the clinical and radiomics variables on the time until recurrence in a cohort of 106 patients, with 30 experiencing the event of interest. The prognostic models provided insights into the predictive power of various factors in Cox proportional hazards frameworks. The clinical model revealed significant predictors such as grade, smoker status (*p* = 0.029), and CIS (*p* = 0.027). The radiomics model highlighted wavelet.LLH_glrlm_RunEntropy as a significant predictor (*p* = 0.023). The combined model emphasized the importance of original¬_glcm_ClusterTendency (Exp (Coef) = 1.499, *p* = 0.011; Supplementary Material S1). These findings demonstrated the utility of assembling clinical and radiomics data to enhance the predictive accuracy of patient outcomes (Table 4).

### 3.7. Assessment of Combined Clinico-Radiomics Model for UTUC Recurrence Prediction

**Figure 5:**
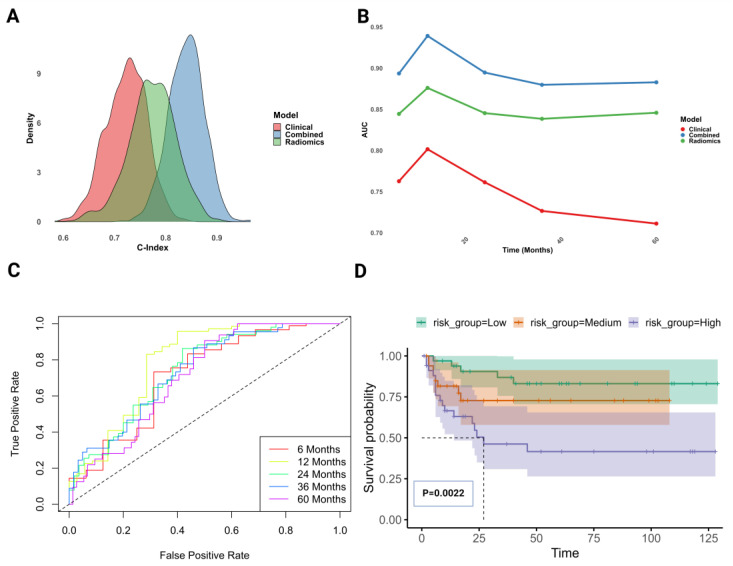
Comparing the performance of clinical and radiomics data combinations for enhanced prognostic accuracy in UTUC recurrence. (**A**) Distribution of C-indices across bootstrap samples, demonstrating the superior performance of the combined model compared to the clinical and radiomics models; (**B**) analysis of the AUC over time for the combined, clinical, and radiomics models demonstrated the consistent accuracy of the combined model over 60 months, underscoring the effectiveness of data integration in enhancing predictive accuracy; (**C**) ROC curves for the combined model at different time points showed the best accuracy in predicting; (**D**) KM survival analysis and risk stratification demonstrated the combined model’s significant (*p* < 0.05) discriminative power in assessing survival outcomes.

This comprehensive statistical evaluation validated the predictive efficacy of three Cox proportional hazards models—clinical, radiomics, and combined datasets—using bootstrap resampling regarding patient recurrence times. The clinical model exhibited moderate predictive accuracy with a C-index of 0.7228 (95% CI: 0.6398–0.7942). The radiomics model demonstrated greater efficacy, achieving a C-index of 0.7736 (95% CI: 0.6684–0.8623). The combined model, merging clinical and radiomics data, achieved a superior performance with the highest predictive accuracy, displaying a C-index of 0.8402 (95% CI: 0.7698–0.9053; Figure 5A). The combined model demonstrated the best fit, as noted by the lowest AIC and BIC values of 232.4837 and 249.4418, respectively.

Over 60 months, model performance variability was observed, with the combined model exhibiting the most stable predictive accuracy, underscoring the effectiveness of data convergence for enhancing the survival analysis predictive performance (Figure 5B). The AUC-ROC evaluation revealed that the combined model achieved the highest prognostic accuracy, with AUC ranging from 0.880 (60 months) to 0.939 (12 months), highlighting the substantial enhancement afforded by combining clinical and radiomics predictors (Figure 5C). The survival outcomes were assessed using the KM method and risk stratification (Figure 5D), with the combined model demonstrating significant discriminative power (*p* = 0.0022; Supplementary Material S1).

## 4. Discussion

This study demonstrated the potential of integrating radiomics features derived from CT urography with clinical factors to enhance the prognostic accuracy in UTUC. Our findings showed that the combined clinico-radiomics model outperformed traditional clinical models in predicting both the survival and recurrence outcomes in UTUC, representing a step forward in personalized medicine. The superior performance of the combined model compared to the clinical and radiomics models for survival prediction underscored the value of integrating diverse data sources. This improvement in the prognostic accuracy could significantly impact clinical decision-making, potentially leading to more personalized treatment strategies in UTUC.

The enhanced predictive power could aid clinicians in stratifying patients more accurately, potentially leading to more appropriate treatment selection and improved patient outcomes. Notably, in the combined survival model, the smoking status emerged as a significant predictor with a counterintuitive protective effect and warranted further investigation into the contributory role of smoking in UTUC outcomes. Our analysis identified specific radiomics features, such as wavelet.LHL_glcm_Correlation and wavelet.LLH_glcm_InverseVariance, as significant predictors of survival. These texture-based features, which captured complex patterns of tumor heterogeneity, likely reflected underlying biological characteristics [42].

Evaluation across various time intervals (12, 24, 36, and 60 months) showed that while clinical and radiomics models displayed varied performance, the combined model offered consistent and precise prognostication. The AUC-ROC analysis revealed its robust predictive capability over time, while the KM analysis demonstrated statistically significant survival probability differences favoring the combined model (*p* < 0.0001).

The combined model again demonstrated superior performance for recurrence prediction compared to the clinical and radiomics models. The higher AUC observed for the combined model further validated its prognostic power. Identifying original_glcm_ClusterTendency as a significant predictor in the combined recurrence model suggested that certain textural features may be particularly relevant for assessing the recurrence risk. Interestingly, in the recurrence model, the smoking status was associated with a significantly higher risk of recurrence, contrasting its impact on survival. Additionally, CIS was identified as a strong predictor of recurrence, highlighting its importance as a prognostic factor. This could have important implications for post-treatment surveillance strategies and early intervention in high-risk patients.

The sub-analysis comparing models with and without recurrence and CIS as predictors revealed intriguing insights into the complex interplay between clinical and radiomics features. While the inclusion of these factors slightly allayed the model’s overall performance, it highlighted different radiomics features as significant predictors. The shift in significant features (from wavelet.LLH_glcm_InverseVariance and wavelet.LHL_glcm_Correlation in the main analysis to wavelet.HLH_glcm_Correlation in the sub-analysis) suggested that the prognostic value of specific radiomics features may depend on the clinical context. This underscores the importance of careful feature selection in model development and highlights the need for context-specific prognostic tools.

The persistence of wavelet-based features across diverse analyses indicated their robust prognostic value, potentially capturing the underlying tumor heterogeneity that remains relevant across different clinical contexts. The emergence of original_gldm_SmallDependenceLowGrayLevelEmphasis as a significant predictor in the sub-analysis implied that certain texture features may become more relevant when considering recurrence and CIS. Such features might capture subtle (image) characteristics associated with aggressive tumor behavior that were more apparent in the context of these high-risk clinical factors.

Despite the inclusion of additional prognostic factors, the observed decrease in model performance in the sub-analysis is an intriguing finding that warrants further investigation. Several factors may contribute to this phenomenon, including aggravated model complexity due to the inclusion of recurrence and CIS, which might have masked the subtle prognostic information provided by radiomics features, and complex interactions with other variables, including radiomics features, in conjunction with the limited sample size of this study. The sub-analysis may be more susceptible to these issues.

To address the challenges posed by the limited dataset, we employed several methodological strategies to boost the model reliability and avoid overfitting. These included k-fold cross-validation, regularization methods, early stopping, feature selection, and hyperparameter tuning. These measures significantly curtailed the risk of overfitting and set the stage for future studies with external dataset validations. Using these techniques enhanced the robustness of our findings and increased the confidence in the models.

While our study focused on CT-based radiomics, it is important to consider other emerging approaches in UTUC prognosis and diagnosis. Recent advancements in the genetic analysis of urothelial cancer have shown promise in enhancing our understanding of tumor biology and potentially guiding treatment decisions. A recent study conducted a transcriptomic analysis in patients with muscle-invasive bladder cancer and identified key genetic signatures associated with disease progression [43]. Although this study dealt with bladder cancer, similar genetic approaches could be applied to UTUC, potentially complementing radiomics-based prognostic models.

Moreover, the role of urine cytology and DNA methylation analysis in UTUC diagnosis and prognosis should not be overlooked. A comparative study of urine cytology and DNA methylation analysis in urinary samples for detecting upper urothelial tract high-grade carcinoma suggested that DNA methylation analysis may offer improved sensitivity compared to traditional urine cytology [13]. Integrating such molecular markers with radiomics features could further enhance the accuracy of UTUC prognostic models and potentially provide insights into the molecular mechanisms underlying the pathology.

The emergence of radiomics analysis, driven by advancements in biomedical imaging and machine learning, is poised to set a paradigm shift in oncotherapeutics [44]. The tumor microenvironment is a fast-evolving, unpredictable biological tissue matrix influenced by immunomodulatory agents enabling cancer cells to evade immune recognition. Many molecular events are only noticeable at a microscopic scale and may be missed during clinical examination [45,46]. Current textural analysis protocols, catalyzed by powerful imaging units, can spot minuscule changes in the tumor microenvironment, especially at the tissue matrix level. When combined with clinical findings, this presents a more inclusive and rationally justifiable model for understanding tumors.

The ability of radiomics to capture subtle changes in the tumor microenvironment may explain its additive prognostic value when combined with clinical factors. The radiomics features may detect early signs of treatment response or resistance or identify patterns associated with more aggressive tumor behavior before they become clinically apparent. Radiomics models have been successfully employed in various cancer types to assess mortality risk. However, determining the optimal algorithm for a given application remains challenging [47,48,49,50].

This study also has certain limitations. Its retrospective nature and relatively small sample size may limit generalizability. Future studies with larger, multi-institutional cohorts are needed to validate these findings. Additionally, while our models showed good discriminative power, their clinical utility needs to be checked to see whether their use leads to improved patient outcomes.

## 5. Conclusions

This study demonstrated the potential of combining CT-based radiomics features with clinical factors to enhance the prognostic accuracy in UTUC. The superior performance of the combined model for both survival and recurrence prediction suggested that this approach could lead to more precise risk stratification and the design of personalized treatment strategies. Such models may further enhance the current management protocols for UTUC by aiding clinicians in decision-making and empowering patients to make treatment choices. Furthermore, it opens several avenues for future research. The prospective validation of these models in different clinical settings and populations is crucial. Exploring the causal relationships between radiomics features and tumor biology could provide insights into the underlying mechanisms of UTUC progression. Combining other imaging modalities, such as PET/CT or advanced MRI techniques, with radiomics analysis may enhance the prognostic accuracy. Integrating radiomics with other biomarkers, including circulating tumor DNA or novel urine-based markers, represents an exciting frontier to explore.

## Figures and Tables

**Table 1:** Overview of patient characteristics and tumor features in the entire cohort (n = 106). [#:count; CTU, CT urogram].

Clinical Variables	Entire Cohort (n = 106)
Age—mean age; age range	74 years (49–93 years)
Gender # (%)	
Female	44 (41%)
Male	62 (58%)
Smoker # (%)	
Never	25 (23%)
Smoker	32 (30%)
Ex-Smoker	49 (46%)
BMI # (%)	
Normal range	35 (33%)
Overweight (pre-obese)	36 (34%)
Obese	35 (33%)
Cytology # (%)	
Positive	22 (20%)
Negative	31 (29%)
Suspicious	10 (9%)
Nondiagnostic	43 (40%)
Deceased, yes, # (%)	58 (54%)
Metastasis, yes, # (%)	12 (11%)
CTU findings positive, yes, # (%)	106 (100%)
Laterality # (%)	
Left	43 (40%)
Right	63 (59%)
Location # (%)	
Renal Pelvis	60 (56%)
Ureter	66 (62%)
Histological grade # (%)	
Low grade	31 (29%)
High grade	75 (70%)
T stage # (%)	
T1	59 (55%)
T2	19 (17%)
T3 or T4	28 (26%)
Carcinoma in situ, yes, # (%)	25 (23%)
Hydronephrosis, yes, # (%)	25 (23%)
Multifocal, yes, # (%)	38 (35%)
Tumor size (cm)	
Mean (standard deviation)	1.97 (0.83)
Median (interquartile range)	1.87 (1.42–2.36)
Range	0.63–5.32
Survival, yes, # (%)	47 (44%)
Recurrence, yes, # (%)	31 (29%)

**Table 2:** Performance of classification models of multivariate Cox regression analysis of clinical, radiomics, and combined datasets to determine the prognostic significance of specific features for survival in UTUC. Significant data points (*p* < 0.05) are marked in bold fonts. [Abbreviations: BMI, body mass index; CI, confidence interval; Exp. Coef., exponential of coefficients].

Models	Variables	Coefficient	Exp. Coef.	Std. Error	Z	*p*-Value	95% CI
Clinical	Tumor size	0.74	2.11	0.34	2.19	**0.02**	2.95–62.19
	Size	−0.40	0.66	0.27	−1.44	0.14	0.38–1.15
	Grade	−0.004	0.99	0.19	−0.02	0.98	0.68–1.45
	Smoker	0.59	1.82	0.46	1.30	0.19	0.73–4.49
	Cytology	−0.39	0.67	0.19	−2.03	**0.04**	0.46–0.98
	Metastasis	−0.63	0.53	0.39	−1.61	0.10	0.24–1.14
	Hydronephrosis	0.61	1.85	0.69	0.89	0.37	0.47–7.18
	BMI	−0.84	0.42	0.40	−2.10	**0.03**	0.19–0.94
	Stage	0.05	1.05	0.03	1.71	0.08	0.99–1.12
	Multifocal	−0.96	0.38	0.44	−2.17	**0.02**	0.16–0.90
	Location	−0.08	0.91	0.38	−0.22	0.82	0.43–1.93
	Gender	−0.02	0.97	0.20	−0.10	0.91	0.65–1.45
	Age	−0.17	0.83	0.17	−0.99	0.32	0.58–1.18
Radiomics	original_glcm_Correlation	−0.15	0.85	0.55	−0.28	0.77	1.33–12.87
	original_glcm_MCC	0.23	1.26	0.56	0.41	0.6	1.51–44.46
	gradient_glcm_Correlation	−0.59	0.55	0.50	−1.17	0.23	1.22–4.41
	gradient_glcm_MCC	0.49	1.64	0.43	1.15	0.24	2.02–45.40
	squareroot_firstorder_Kurtosis	0.21	1.23	0.21	0.97	0.32	2.24–6.63
	wavelet.LHL_glcm_Correlation	0.73	2.09	0.40	1.84	**0.06**	2.59–98.60
	wavelet.HHL_glcm_Correlation	−0.20	0.81	0.47	−0.43	0.66	1.37–7.98
	wavelet.HHH_glcm_Imc1	0.25	1.28	0.24	1.02	0.30	2.20–8.11
	wavelet.HHH_glcm_Correlation	0.10	1.11	0.33	0.32	0.74	1.78–8.41
	wavelet.LHL_firstorder_Skewness	−0.10	0.89	0.13	−0.83	0.40	1.99–3.18
Combined	Smoker	−0.39	0.67	0.18	−2.12	**0.03**	0.47–0.96
	Hydronephrosis	−0.07	0.93	0.38	−0.18	0.85	0.44–1.97
	wavelet.LLH_glcm_InverseVariance	0.56	1.75	0.24	2.28	0.02	1.08–2.84
	wavelet.LHL_glcm_Correlation	0.58	1.78	0.15	3.80	0.0001	1.32–2.41
	wavelet.HHL_glcm_Correlation	0.29	1.34	0.24	1.17	0.24	0.82–2.18
	wavelet.HHH_glcm_Correlation	0.41	1.51	0.31	1.33	0.18	0.82–2.80
	wavelet.LHH_glcm_Imc2	0.09	1.10	0.34	0.28	0.77	0.55–2.18

**Table 3:** Performance of the Cox proportional hazards combined models for main analysis (excluding recurrence and CIS) and sub-analysis (including recurrence and CIS) to assess the prognostic significance of specific features in predicting survival outcomes in UTUC. Significant data points (*p* < 0.05) are marked in bold font. [Abbreviations: CI, confidence interval; Exp. Coef., exponential of coefficients; Std. Error, standard error].

Models	Variables	Coefficient	Exp. Coef.	Std. Error	Z	*p*-Value	95% CI
Main analysis	Smoker	−0.391	0.67	0.18	−2.12	**0.03**	0.47–0.96
	Hydronephrosis	−0.07	0.93	0.38	−0.184	0.85	0.44–1.97
	wavelet.LLH_glcm_InverseVariance	0.56	1.75	0.24	2.28	**0.023**	1.08–2.84
	wavelet.LHL_glcm_Correlation	0.58	1.79	0.15	3.8	**0.0001**	1.32–2.41
Sub-analysis	Hydronephrosis	−1.17	0.31	0.82	−1.42	0.15	0.06–1.55
	original_shape_Elongation	−0.78	0.45	0.41	−1.86	0.06	0.2–1.03
	original_shape_Sphericity	0.26	1.29	0.34	0.75	0.44	0.66–2.55
	original_gldm_SmallDependenceLowGrayLevelEmphasis	1.11	3.05	0.4	2.74	**0.006**	1.37–6.77
	gradient_glcm_Correlation	0.7	2.02	0.43	1.62	0.1	0.86–4.72
	squareroot_firstorder_Kurtosis	0.24	1.27	0.49	0.49	0.61	0.48–3.33
	wavelet.LHL_glcm_Idmn	−0.02	0.97	0.4	−0.06	0.94	0.43–2.16
	wavelet.HLL_gldm_SmallDependenceLowGrayLevelEmphasis	−0.29	0.74	0.32	−0.9	0.36	0.39–1.4
	wavelet.HLH_glcm_Correlation	1.01	2.75	0.45	2.24	**0.02**	1.136–6.7
	wavelet.HLH_glcm_Imc1	0.56	1.75	0.32	1.71	0.08	0.92–3.33
	wavelet.HLH_glcm_InverseVariance	0.696	2.007	0.48	1.43	0.15	0.77–5.19

**Table 4:** Performance of classification models of multivariate Cox regression analysis of clinical, radiomics, and combined datasets to determine the prognostic significance of specific features for recurrence in UTUC. Significant data points (*p* < 0.05) are marked in bold fonts. [Abbreviations: CI, confidence interval; Exp. Coef., exponential of coefficients; Std. Error, standard error].

Models	Variables	Coefficient	Exp. Coef.	Std. Error	Z	*p*-Value	95% CI
Clinical	Grade	0.86	2.36	0.49	1.72	0.08	0.89–6.27
	Smoker	0.71	2.59	0.23	2.18	**0.02**	1.37–7.94
	CIS	0.87	2.39	0.39	2.19	**0.02**	1.09–5.20
Radiomics	original_shape_Elongation	0.13	1.14	0.26	0.49	0.61	0.67–1.91
	original_shape_MajorAxisLength	0.31	1.36	0.24	1.29	0.19	0.85–2.19
	original_shape_Sphericity	−0.02	0.97	0.24	−0.11	0.90	0.60–1.56
	original_glcm_ClusterTendency	0.35	1.42	0.20	1.71	0.08	0.95–2.12
	original_gldm_LowGrayLevelEmphasis	−0.42	0.65	0.27	−1.55	0.11	0.38–1.11
	wavelet.LLH_firstorder_Mean	−0.57	0.56	0.33	−1.74	0.08	0.29–1.07
	wavelet.LLH_glrlm_RunEntropy	−0.69	0.49	0.30	−2.26	**0.02**	0.27–0.91
Combined	Grade	0.80	2.22	0.50	1.59	0.11	0.83–5.97
	Smoker	0.45	0.63	0.26	1.74	0.08	0.37–1.05
	CIS	0.75	2.12	0.41	1.83	0.06	0.94–4.78
	original_shape_Elongation	−0.15	0.85	0.25	−0.61	0.53	0.51–1.41
	original_shape_MajorAxisLength	0.24	1.27	0.24	1.00	0.31	0.79–2.04
	original_shape_Sphericity	−0.03	0.96	0.23	−0.16	0.86	0.60–1.53
	original_glcm_ClusterTendency	0.40	1.49	0.16	2.52	**0.01**	1.09–2.05

## Data Availability

All the data are reported within the manuscript. Further information and clarification may be obtained from the corresponding author (G.N.) upon reasonable request.

## Supplementary Materials

The following supporting information can be downloaded at https://www.mdpi.com/article/10.3390/cancers16183119/s1, Supplementary Material S1. Patients and methods—detailed overview.
