# Supplementary material for: Radiomics-Based Computed Tomography Urogram Approach for the Prediction of Survival and Recurrence in Upper Urinary Tract Urothelial Carcinoma
Source: Cancers (Basel). 2024 Sep 10;16(18):3119. doi: 10.3390/cancers16183119 (PMC11429600; doi:10.3390/cancers16183119)
Supplement: Supplementary file 1 [file cancers-16-03119-s001.zip › cancers-3152647-supplementary.pdf]

SUPPLEMENTARY MATERIALS

Radiomics-based CT urogram approach for the prediction of survival and recurrence in upper urinary tract urothelial carcinoma

Abdulsalam Alqahtani,[1,2] Sourav Bhattacharjee,[3]
Abdulrahman Almopti,[1] Chunhui Li,[4] Ghulam Nabi[1,*]

[1]School of Medicine, Centre for Medical Engineering and Technology, University of Dundee, Dundee DD1 9SY, UK

[2]Radiology Department, College of Applied Medical Sciences, Najran University, Najran 55461, Kingdom of Saudi Arabia

[3]School of Veterinary Medicine, University College Dublin, Belfield, Dublin 4, Ireland

[4]School of Science and Engineering, University of Dundee, Dundee DD1 4HN, UK

*Corresponding author

Email: GNabi@dundee.ac.uk; T.: +44 (0)1382 383192

ORCiD

Abdulsalam Alqahtani (AAQ): https://orcid.org/0000-0001-7243-8042

Sourav Bhattacharjee (SB): https://orcid.org/0000-0002-6528-6877

Abdulrahman Almopti (AAM): https://orcid.org/0009-0004-6513-4795

Chunhui Li (CL): https://orcid.org/0000-0003-2186-5137

Ghulam Nabi (GN): https://orcid.org/0000-0001-9406-5195

Supplementary Material S1

Patients and methods—Detailed overview

Comprehensive clinical, radiomics, and combined data analysis for outcome prediction

The univariate Cox proportional hazards regression analysis revealed several significant predictors of patient outcomes. Notably, the presence of recurrence (Estimate: 0.7103, p=0.0344, Exp (Coef): 2.0347) and carcinoma *in situ* (CIS) (Estimate: 0.7696, p=0.0251, Exp(Coef): 2.1590) were associated with a higher HR, suggesting a substantial impact on patient prognosis. Tumor size (Estimate: –0.4126, p=0.0927, Exp (Coef): 0.6619) demonstrated a trend toward attaining significance (p<0.05), indicating that smaller tumor size might be associated with better outcomes. Among radiomics features, original_glcm_Correlation (Estimate: 0.4970, p=0.0011, Exp (Coef): 1.6437), original_glcm_MCC (Estimate: 0.4981, p=0.0019, Exp(Coef): 1.6456), and gradient_glcm_Correlation (Estimate: 0.4725, p=0.0110, Exp(Coef): 1.6040) were significant, highlighting the potential of these imaging biomarkers in predicting patient survival. Additionally, several wavelet-transformed radiomics features, such as wavelet.HHL_glcm_Correlation (Estimate: 0.5769, p<0.0001, Exp (Coef): 1.7805) and wavelet.LHL_glcm_Correlation (Estimate: 0.5974, p<0.0001, Exp (Coef): 1.8174), were (strongly) associated with outcomes. Conversely, features such as smoker status (Estimate: –0.2531, p=0.1581, Exp (Coef): 0.7764) and age at operation (Estimate: –0.0428, P=0.8068, Exp (Coef): 0.9581) did not show significant associations (Table S1).

Table S1. A comprehensive univariate Cox regression analysis of clinical, radiomics, and combined datasets to determine the prognostic significance of specific features in UTUC.

Variable	Estimate	Std_Error	Z	p–value	Exp_Coef	Lower_CI	Upper_CI
T_size	–0.4126	0.2454	–1.6811	0.0927	0.6619	1.5055	2.9178
Grade	0.1501	0.3082	0.4871	0.6262	1.162	1.8873	8.3798
Smoker	–0.2531	0.1793	–1.4115	0.1581	0.7764	1.727	3.0142
cytology	–0.0476	0.2948	–0.1615	0.8717	0.9535	1.7076	5.4691
Metastasis	0.2927	0.5267	0.5558	0.5784	1.34	1.6118	43.032
Hydronephrosis	–0.5247	0.3597	–1.4588	0.1446	0.5917	1.3396	3.3119
Body. Mass. index	0.0136	0.0301	0.4536	0.6501	1.0137	2.6006	2.9307
Stage	–0.4883	0.322	–1.5166	0.1294	0.6137	1.3861	3.169
Multifocal	0.1157	0.3052	0.3789	0.7048	1.1226	1.8537	7.7051
Location	–0.026	0.1504	–0.1726	0.863	0.9744	2.0661	3.6999
Side	0.0381	0.1485	0.2568	0.7973	1.0389	2.1741	4.0136
Gender	–0.1162	0.1502	–0.7738	0.439	0.8903	1.9411	3.3036
Age at operation	–0.0428	0.1749	–0.2445	0.8068	0.9581	1.974	3.8572
original_shape_Elongation	0.0207	0.1491	0.1385	0.8898	1.0209	2.1428	3.9254
original_shape_LeastAxisLength	–0.2984	0.1991	–1.499	0.1339	0.742	1.6524	2.9926
original_shape_MajorAxisLength	–0.1494	0.1587	–0.9416	0.3464	0.8612	1.8794	3.2395
original_shape_Maximum2DDiameterColumn	–0.0975	0.1567	–0.6224	0.5337	0.9071	1.9489	3.4319
original_shape_Maximum2DDiameterSlice	–0.1078	0.1997	–0.5401	0.5891	0.8978	1.835	3.7726
original_shape_MeshVolume	–0.559	0.3954	–1.4137	0.1575	0.5718	1.3014	3.4594
original_shape_Sphericity	–0.0731	0.163	–0.4484	0.6539	0.9295	1.9648	3.5942
original_shape_SurfaceVolumeRatio	0.2808	0.1523	1.844	0.0652	1.3242	2.6711	5.9587
original_glcm_Correlation	0.497	0.1526	3.2576	**0.0011**	1.6437	3.3835	9.1758

original_glcm_Idn	0.4571	0.1688	2.7074	**0.0068**	1.5795	3.1096	9.0154
original_glcm_Imc1	–0.3802	0.1515	–2.5101	**0.0121**	0.6837	1.6621	2.5094
original_glcm_MCC	0.4981	0.1606	3.1008	**0.0019**	1.6456	3.3239	9.5315
original_gldm_SmallDependenceLowGrayLevelEmphasis	0.0118	0.1577	0.0746	0.9406	1.0118	2.1019	3.9677
original_glszm_SmallAreaEmphasis	–0.0712	0.1252	–0.5685	0.5697	0.9313	2.0724	3.2879
original_ngtdm_Coarseness	0.2582	0.1309	1.9731	**0.0485**	1.2946	2.723	5.3293
gradient_glcm_Correlation	0.4725	0.1858	2.5428	**0.011**	1.604	3.0477	10.0603
gradient_glcm_MCC	0.4368	0.1616	2.703	**0.0069**	1.5477	3.0882	8.3674
squareroot_firstorder_Kurtosis	0.2396	0.1059	2.2627	**0.0237**	1.2708	2.8083	4.7773
squareroot_firstorder_Skewness	0.1968	0.1507	1.3058	0.1916	1.2175	2.4747	5.1336
wavelet.LLH_firstorder_90Percentile	0.0072	0.1664	0.0435	0.9653	1.0073	2.0687	4.038
wavelet.LLH_glcm_Correlation	0.1914	0.1361	1.406	0.1597	1.2109	2.5278	4.8609
wavelet.LLH_glcm_Imc1	–0.2058	0.1837	–1.1199	0.2628	0.814	1.7645	3.212
wavelet.LLH_glcm_Imc2	0.2587	0.1796	1.4401	0.1498	1.2953	2.4864	6.3089
wavelet.LLH_glcm_InverseVariance	0.4213	0.2188	1.9254	0.0542	1.524	2.6979	10.3817
wavelet.LLH_glcm_MCC	0.2993	0.1709	1.7507	0.08	1.3489	2.6244	6.5912
wavelet.LLH_gldm_LargeDependenceEmphasis	–0.0411	0.1535	–0.2678	0.7889	0.9597	2.0347	3.6569
wavelet.LLH_gldm_LargeDependenceLowGrayLevelEmphasis	–0.2346	0.1784	–1.3152	0.1884	0.7909	1.7463	3.0707
wavelet.LLH_glszm_LargeAreaEmphasis	–0.3868	0.3877	–0.9976	0.3184	0.6792	1.3739	4.2727
wavelet.LHL_firstorder_Skewness	0.0888	0.1273	0.6977	0.4854	1.0928	2.3434	4.065
wavelet.LHL_glcm_Correlation	0.5974	0.1414	4.2235	0	1.8174	3.9644	11.0006
wavelet.LHL_glcm_Idmn	0.2719	0.1756	1.5487	0.1215	1.3124	2.5354	6.3689
wavelet.LHL_glcm_Imc2	–0.1276	0.1747	–0.7305	0.4651	0.8802	1.8682	3.4542
wavelet.LHL_glcm_MCC	–0.0791	0.1663	–0.4758	0.6342	0.9239	1.9483	3.5962
wavelet.LHL_glrlm_RunLengthNonUniformityNormalized	–0.3736	0.1795	–2.0809	**0.0374**	0.6882	1.6227	2.6605
wavelet.LHL_glszm_ZoneEntropy	–0.3593	0.1419	–2.5327	**0.0113**	0.6982	1.6967	2.5142
wavelet.LHH_glcm_Correlation	0.4232	0.1558	2.7167	**0.0066**	1.5268	3.0806	7.9405
wavelet.LHH_glcm_Imc1	0.1907	0.205	0.9305	0.3521	1.2101	2.2474	6.1004
wavelet.LHH_glcm_Imc2	–0.4721	0.2081	–2.2679	**0.0233**	0.6237	1.514	2.5546
wavelet.LHH_glcm_MCC	–0.4614	0.2073	–2.2256	**0.026**	0.6304	1.5218	2.5765
wavelet.HLL_glcm_Correlation	0.3623	0.1167	3.1045	**0.0019**	1.4366	3.1358	6.0847
wavelet.HLL_glcm_Idn	0.2484	0.1589	1.5633	0.118	1.2819	2.5572	5.7561
wavelet.HLL_glcm_Imc1	–0.2008	0.1143	–1.7568	0.079	0.8181	1.9231	2.7829
wavelet.HLL_glcm_Imc2	0.2381	0.1338	1.7803	0.075	1.2689	2.6545	5.2027
wavelet.HLL_glcm_MCC	0.2976	0.1364	2.1813	**0.0292**	1.3466	2.8029	5.8089
wavelet.HLL_gldm_SmallDependenceLowGrayLevelEmphasis	0.0497	0.1502	0.331	0.7407	1.051	2.1879	4.0992
wavelet.HLL_glrlm_LongRunEmphasis	–0.1011	0.1806	–0.5601	0.5754	0.9038	1.8859	3.6243
wavelet.HLH_firstorder_Kurtosis	0.1407	0.1146	1.2271	0.2198	1.1511	2.5078	4.2252
wavelet.HLH_glcm_Correlation	0.2522	0.1095	2.3028	**0.0213**	1.2869	2.8243	4.9287
wavelet.HLH_glcm_Imc1	0.001	0.1452	0.0069	0.9945	1.001	2.1237	3.7827
wavelet.HLH_glcm_InverseVariance	0.1206	0.1808	0.6668	0.5049	1.1281	2.2067	4.9925
wavelet.HLH_glcm_MCC	–0.0893	0.1968	–0.4534	0.6503	0.9146	1.8624	3.839
wavelet.HHL_glcm_Correlation	0.5769	0.1265	4.5604	0	1.7805	4.0129	9.7915
wavelet.HHL_glcm_Imc1	0.1135	0.1677	0.6772	0.4983	1.1202	2.24	4.7402
wavelet.HHL_glcm_Imc2	–0.2459	0.1906	–1.29	0.1971	0.782	1.713	3.115
wavelet.HHL_glszm_ZoneEntropy	–0.5177	0.1676	–3.0895	**0.002**	0.5959	1.5358	2.2877

wavelet.HHH_firstorder_Skewness	0.1554	0.1706	0.9109	0.3623	1.1681	2.3075	5.1132
wavelet.HHH_glcm_Correlation	0.5279	0.1565	3.3727	**7e–04**	1.6954	3.4816	10.0152
wavelet.HHH_glcm_Imc1	0.5007	0.2077	2.411	**0.0159**	1.6499	2.9988	11.9264
wavelet.HHH_glcm_MCC	–0.5878	0.2157	–2.7256	**0.0064**	0.5555	1.4391	2.3345
wavelet.LLL_firstorder_Skewness	0.2109	0.1723	1.224	0.2209	1.2348	2.4131	5.6454
wavelet.LLL_glcm_Correlation	0.2219	0.1595	1.3913	0.1641	1.2485	2.4925	5.5109
wavelet.LLL_glcm_Idmn	0.1895	0.1656	1.1445	0.2524	1.2087	2.3957	5.323
wavelet.LLL_glcm_MCC	0.3272	0.1601	2.0432	**0.041**	1.3871	2.755	6.6755
wavelet.LLL_gldm_LargeDependenceHighGrayLevelEmphasis	0.112	0.1785	0.6274	0.5304	1.1185	2.1998	4.8881
wavelet.LLL_gldm_SmallDependenceLowGrayLevelEmphasis	0.1717	0.1417	1.212	0.2255	1.1874	2.4582	4.7948

Evaluating model efficacy and complexity in clinical and radiomics data integration

Here, the application of LASSO Cox regression to clinical (Figure S1A), radiomics (Figure S1B), and combined (Figure S1C) datasets elucidated vital prognostic indicators for patient survival. Cox proportional hazards regression analyses were conducted on the three datasets to identify significant predictors of survival time. The analysis of the clinical dataset identified three variables—hydronephrosis, smoking status, and stage—as potential predictors of survival. Although none of these variables demonstrated statistical significance, hydronephrosis (HR: 0.518, p=0.0708), smoking (HR: 0.715, p=0.0616), and stage (HR: 0.565, p=0.0791)—all exhibited trends suggesting their potential influence on survival. In the radiomics dataset, no single radiomics feature emerged as a statistically significant predictor of survival. However, the model did show that features like wavelet.LHL_glcm_Correlation (HR: 2.093, p=0.0654) approaching significance. The model's C-index was 0.73, suggesting that the radiomics data might provide a better predictive capacity than clinical data alone.

When clinical and radiomics features were integrated, the combined model's predictive performance improved, as reflected by a C-index of 0.737. In this combined model, smoking (HR: 0.597, p=0.009) and the radiomics feature wavelet.LLH_glcm_InverseVariance (HR: 1.853, p=0.024) were identified as significant predictors of survival. The application of LASSO Cox regression and traditional Cox proportional hazards regression to clinical, radiomics, and combined datasets provided valuable insights into the predictors of survival time. The LASSO Cox regression identified key variables within each dataset, with the clinical model highlighting hydronephrosis, smoker, and stage, while the radiomics model emphasized wavelet.LHL_glcm_Correlation and wavelet.HHL_glcm_Correlation as significant features.

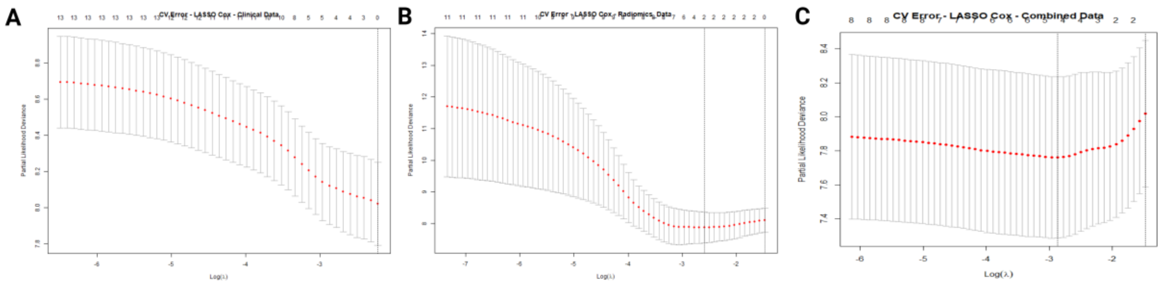

Figure S1. The cross-validation errors for model selection across multiple datasets: (A) clinical, (B) radiomics, and (C) combined. Lower scores indicate better model performance and *vice versa*. The radiomics data has a broader range of λ values, reflecting its complexity, while the combined data provided a middle ground.

Evaluation of Cox proportional hazards regression models across distinctive datasets

In a detailed analysis of Cox proportional hazards regression models across distinct datasets, significant predictors of patient survival times were identified. HRs and 95% CIs were calculated and visualized for each variable. In the clinical model, smoker (HR=0.714, 95% CI: 0.502–1.017, p=0.0616) and stage (HR=0.565, 95% CI: 0.298–1.069, p=0.0791) showed trends toward significance, although with some uncertainty (Figure S2A). The radiomics model identified wavelet.LHL_glcm_Correlation (HR=1.79, 95% CI: 1.359–2.35, p<0.0001) and wavelet.LLH_glcm_InverseVariance (HR=1.52, 95% CI: 0.972–2.36, p=0.0663) as potentially influential features (Figure S2B). The combined model demonstrated enhanced predictive power, with smoker (HR=0.676, 95% CI: 0.471–0.97, p=0.0335) and certain radiomics features, such as wavelet.LLH_glcm_InverseVariance (HR=1.753, 95% CI: 1.082–2.84, p=0.0226) and wavelet.LHL_glcm_Correlation (HR=1.79, 95% CI: 1.326–2.42, p<0.0001), showing significant contributions (Figure S2C). The combined model had the lowest AIC (304.26) and BIC (311.57), indicating the best fit among the models considered. The radiomics model followed closely with an AIC of 304.98 and BIC of 308.64, while the clinical model had the highest AIC (318.24) and BIC (323.72), suggesting a less favorable fit (Figure S2D).

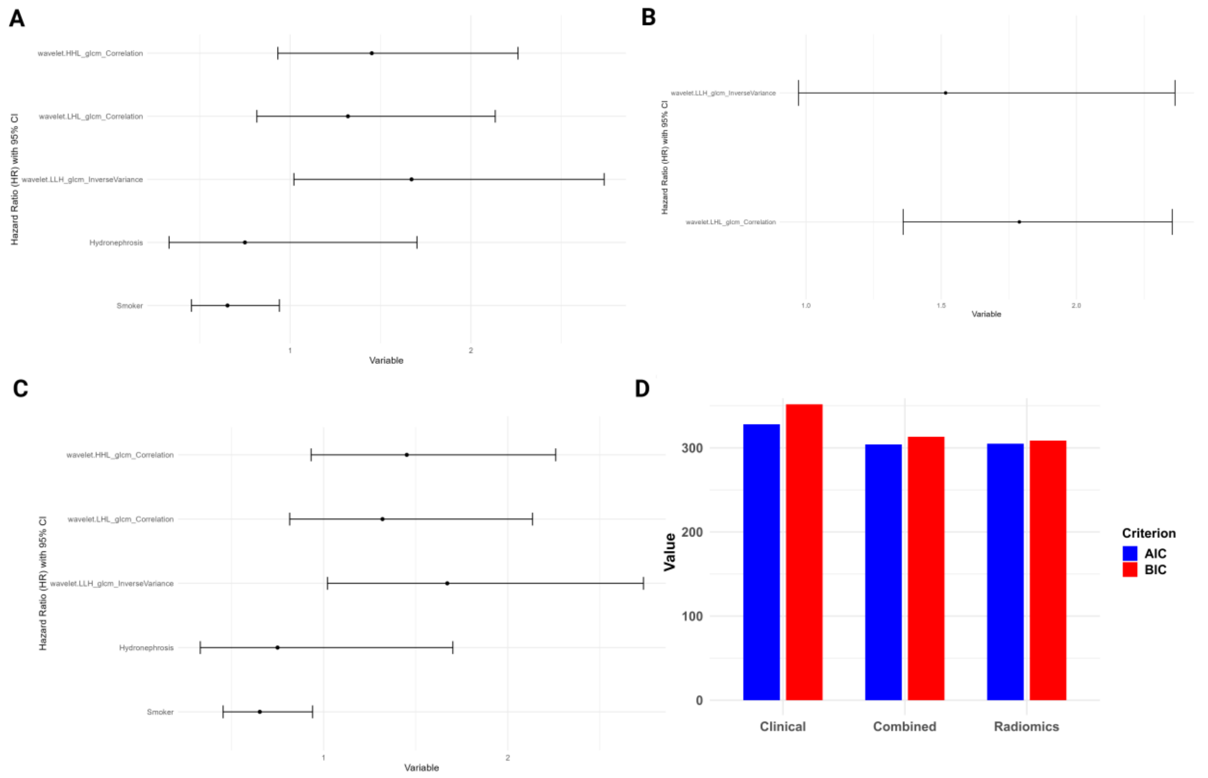

Figure S2. The obtained HRs with 95% CIs for variables from: (A) clinical, (B) radiomics, and (C) combined datasets are shown. (D) The AIC (red) and BIC (blue) values of the three models were plotted.

The interaction analysis conducted between clinical and radiomics variables using the Cox proportional hazards model provides important insights into their relationship with survival outcomes. Specifically, the main effects of key variables such as smoker (HR=0.6431, 95% CI: 0.4408–0.9383, p=0.022) and wavelet.LLH_glcm_InverseVariance (HR=2.5144, 95% CI: 1.1806–5.3553, p=0.0168) were statistically significant, indicating their independent contributions to

the survival prediction model. In contrast, the interaction terms, such as those between smoker and wavelet.LHL_glcm_Correlation (HR=1.0862, 95% CI: 0.7853–1.5024, p=0.6173) and between hydronephrosis and wavelet.LLH_glcm_InverseVariance (HR=0.5279, 95% CI: 0.2050–1.3593, p=0.1855), were not statistically significant. These findings enhance the interpretability of the model by indicating that each variable can be considered separately when predicting outcomes. Furthermore, the model's overall performance, as reflected in a C-index of 0.721, demonstrates its strong predictive capability (Figure S3).

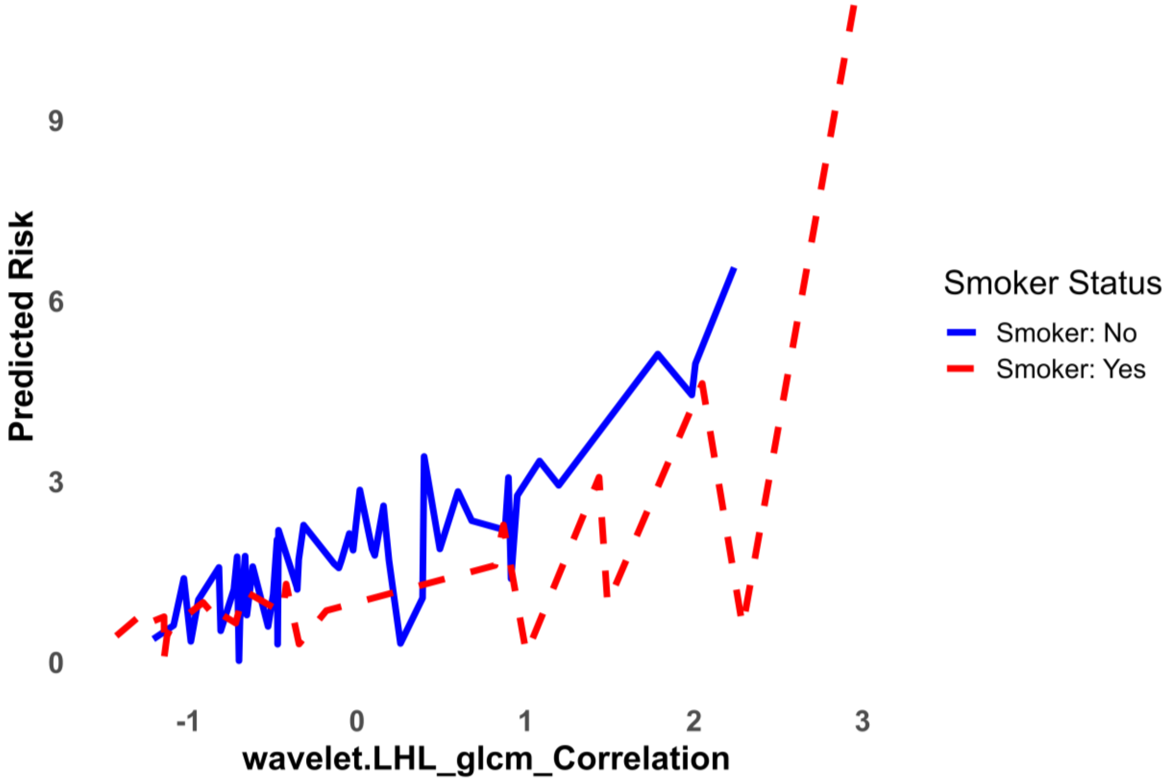

Figure S3. Interaction between smoking status and radiomics feature (wavelet.LHL_glcm_Correlation) on predicted risk. The plot shows the predicted risk of mortality as a function of wavelet.LHL_glcm_Correlation, with separate lines for smokers (red) and non-smokers (blue). The interaction between smoking status and the radiomics feature suggests different risk profiles based on smoking status.

The proportional hazards assumption for the combined clinico-radiomics Cox model was assessed using Schoenfeld residuals, with the results summarized as follows: individual covariates, including smoker (p=0.107), wavelet.LHL_glcm_Correlation (p=0.789), hydronephrosis (p=0.290), and wavelet.LLH_glcm_InverseVariance (p=0.823), all demonstrated insignificant p-values, indicating no significant violation of the proportional hazards assumption for these terms. Additionally, the interaction terms, smoker, wavelet.LHL_glcm_Correlation (p=0.552), hydronephrosis, and wavelet.LLH_glcm_InverseVariance (p=0.095), also did not show significant violations. The global test for the model yielded a χ^2 value of 11.3602 with six degrees of freedom and a p-value of 0.078, suggesting that, overall, the proportional hazards assumption was not significantly violated. However, the global p-value nearing the 0.05 threshold indicated a need for cautious interpretation, particularly regarding the interaction terms and their potential impact on the model validity (Figure S4).

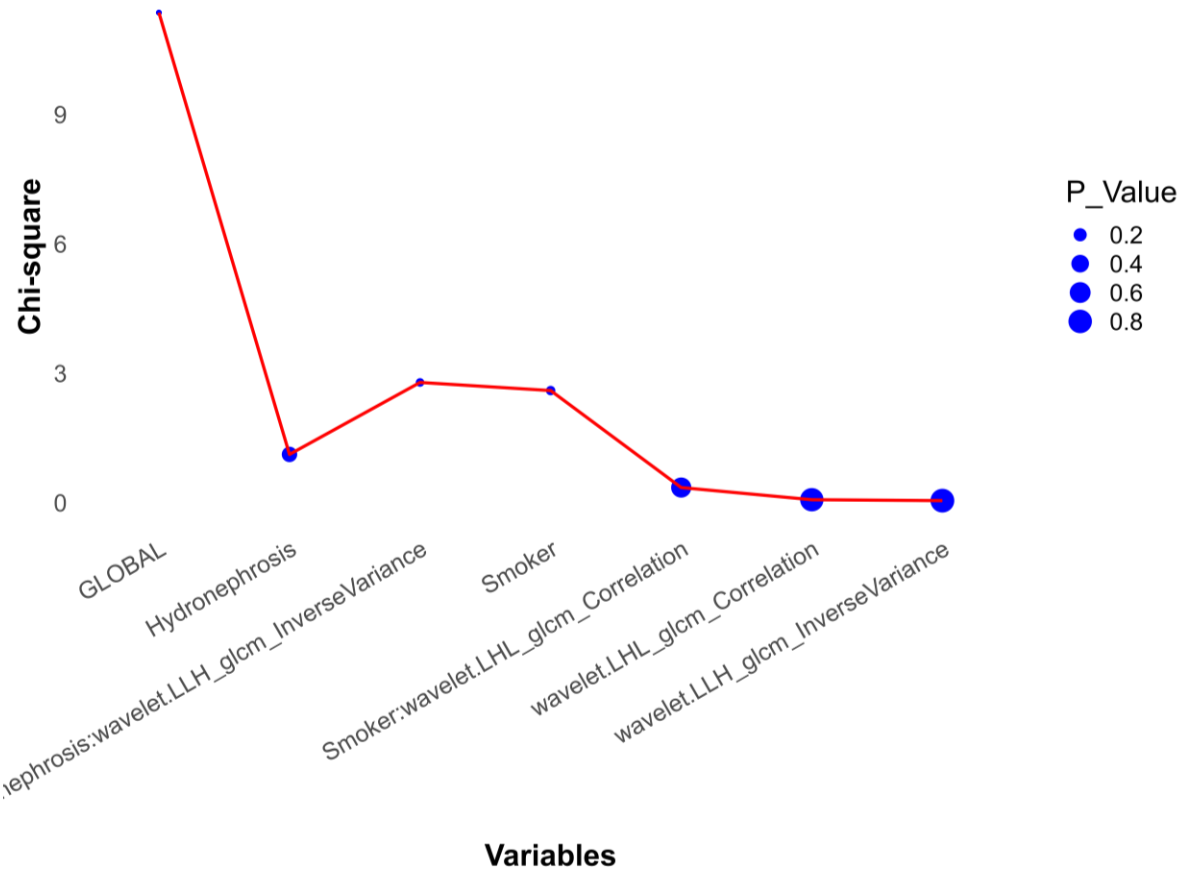

Figure S4. The Schoenfeld residuals analysis for the combined clinico-radiomics Cox model. The individual covariates and interactions demonstrate no significant violations of the proportional hazards assumption ($p>0.05$). The global test ($\chi^2=11.36$, df=6, p=0.078) indicated that the overall model adhered to the proportional hazards assumption, though the global p-value suggested caution when interpreting the interactions, particularly for hydronephrosis and wavelet.LLH_glcm_InverseVariance.

In the combined clinico-radiomics Cox model, the analysis of feature importance revealed that radiomics features, particularly wavelet.LLH_glcm_InverseVariance and wavelet.LHL_glcm_Correlation, played a significant role in predicting survival outcomes. These features exhibited high HRs, with Cis below one, indicating strong and statistically significant associations with the risk of events. In contrast, the clinical feature smoker (Group 1) also showed a meaningful association with survival, although its effect was less pronounced than that of the leading radiomics features. Interaction terms, such as smoker, wavelet.LHL_glcm_Correlation and hydronephrosis, and wavelet.LLH_glcm_InverseVariance, displayed HRs close to one, with wider CIs, suggesting that their effects were less certain and might not be statistically significant. Hydronephrosis itself showed a HR closing one, indicating little to no impact on survival (Figure S5).

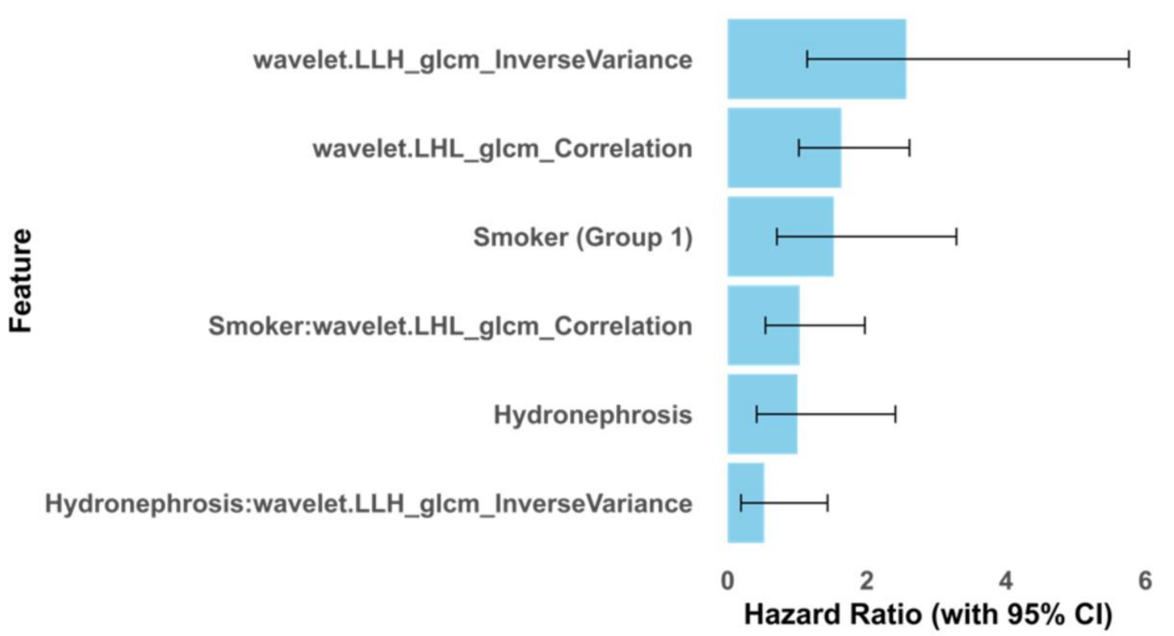

Figure S5. The HRs and corresponding 95% CIs for individual covariates and interaction terms in the combined clinico-radiomics Cox model. The variables, including wavelet.LLH_glcm_InverseVariance, wavelet.LHL_glcm_Correlation, smoker, and their interactions with hydronephrosis, showed the strength and direction of their associations with the outcome. CIs crossing the vertical line at one indicated insignificant effects.

Predictive model evaluation: clinical, radiomics, and combined approaches

The study used the KM survival analysis method to generate survival curves for clinical, radiomics, and combined datasets. These curves allowed for evaluating patient survival probabilities at crucial time points, including 12-, 24-, 36-, and 60-months post-diagnosis or treatment initiation. The results showed an expected downward trend in survival probability over time, characteristic of survival analyses. The quantified survival probabilities and their corresponding 95% CIs provided instrumental cues in clinical decision-making.

A comparable degree of variability was noted while assessing the standard deviations of the model coefficients across all three models. It indicated that the prognostic influence of specific variables on survival outcomes remained variable across the different data types, albeit the combined model slightly decreased the variability. Clinical data had differences in survival rates underscored by p-value of <0.0001 (Figure S6A). Radiomics data had significant differences in survival rates underscored by p-value of <0.0001 (Figures S6B), confirming the superior discriminatory power of the combined model.

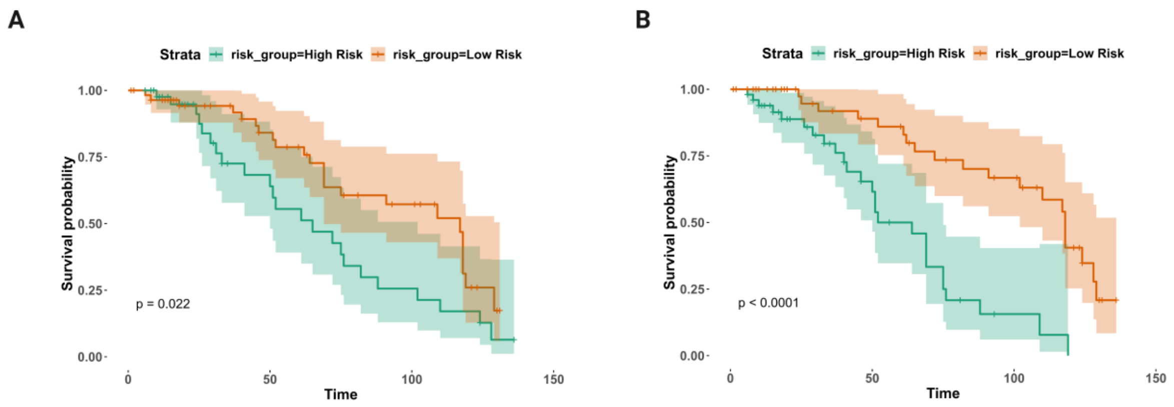

Figure S6. The KM survival curves for predictive models and median survival trajectory. The survival probabilities over time for high-risk (red) and low-risk (green) groups across (A) clinical, and (B) radiomics models over 140 months are shown. The separation between high-risk and low-risk curves illustrated each model's discriminatory power, while the shaded region around the median survival line indicated the 95% CIs.

Prognostic performance over time: clinical versus radiomics and combined dataset predictions

The prognostic performance of Cox regression models across clinical, radiomics, and combined datasets over various time points provided valuable insights into their predictive accuracy for patient outcomes. The AUC and 95% CIs from the ROC analyses were used as evaluation metrics. In this study, the predictive accuracy of Cox proportional hazards models was assessed by calculating the AUCs at 12, 24, 36, and 60 months for clinical and radiomics datasets. For the clinical data, the AUC values were 0.523 at 12 months, 0.575 at 24 months, 0.7459 at 36 months, and 0.6892 at 60 months, indicating a relatively low predictive accuracy at earlier time points with improved performance at 36 and 60 months (Figure S7A). In contrast, the radiomics data showed stronger predictive performance, with AUCs of 0.8301 at 12 months, 0.6949 at 24 months, 0.6786 at 36 months, and 0.7991 at 60 months (Figure S7B).

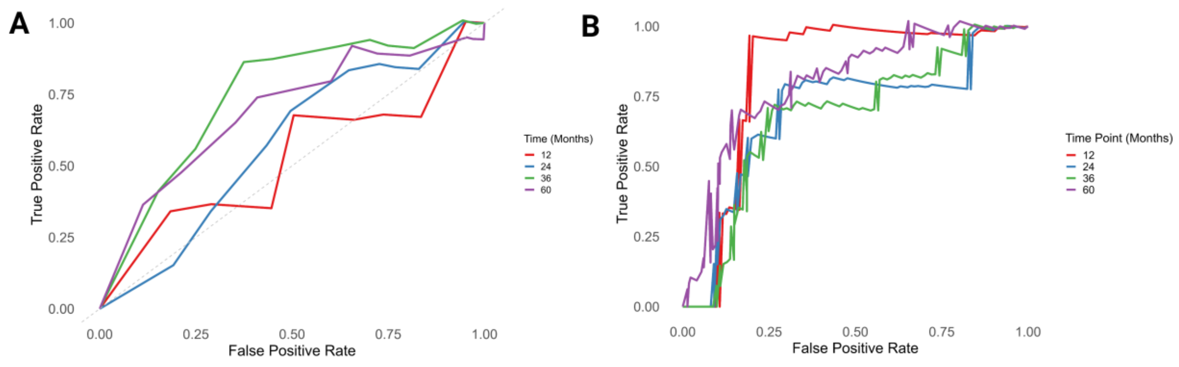

Figure S7. The discriminatory prowess of (A) clinical, and (B) radiomics data-based prognostic models over various time spans.

Table S2: Overview of the performance metrics for clinical, radiomics, and combined models, including C-index, AIC, BIC, and time-dependent AUCs.

Metric	Clinical	Radiomics	Combined
C-index (95% CI)	0.64	0.70	0.75
SE	0.047	0.041	0.037
AIC	318.24	304.98	304.26
BIC	323.72	308.64	311.57
Time-dependent AUCs (95% CI)			
1–year	0.52	0.83	0.71
2–year	0.57	0.69	0.69
3–year	0.74	0.67	0.72
4–year	0.68	0.79	0.84

Recurrence

Predictive variables and their impact on recurrence risk

We conducted a comprehensive statistical analysis to evaluate the predictive value of clinical and radiomics variables on time until recurrence (n=106) of whom 30 experienced the event of interest. The multivariate Cox proportional hazards models were applied to clinical, radiomics and combined datasets to assess the influence of selected variables on time to recurrence. In the clinical model, the variable grade positively correlated with recurrence risk, showing an exponential coefficient of 2.363 and p=0.084. The CIS presented a statistically significant positive correlation with an exponential coefficient of 2.393 (p=0.027). Conversely, smoking history was negatively correlated with recurrence with statistical significance (exponential coefficient of 0.597, p=0.029).

The radiomics model did not yield any statistically significant predictors. Nonetheless, wavelet.LLH.glrlm_RunEntropy displayed a significant negative correlation with recurrence (exponential coefficient of 0.434, p=0.008), and square_glszm_SizeZoneNonUniformityNormalized was similarly negative with significance (exponential coefficient of 0.576, p=0.043).

In the combined model, grade again showed a notable positive association with recurrence (exponential coefficient of 3.405, p=0.022). Other radiomics features, such as wavelet.LLH-_glrlm_RunEntropy, mirrored the negative correlation (exponential coefficient of 0.353, p=0002). Integratedly, these analyses suggested that specific clinical factors, particularly grade and CIS, were consistent indicators of recurrence risk (Table S3).

Table S3. Comparison of the clinical, radiomics, and combined multivariate Cox models.

Models	Variables	Estimate	Exp_Coef.	Std_Error	Z	p-value	Lower_CI	Upper_CI
Multivariate Cox model clinical	Grade	0.860	2.363	0.498	1.726	0.084	1.193	9.720
	Smoker	–0.515	0.597	0.236	–2.182	**0.029**	0.567	1.690
	CIS	0.872	2.393	0.396	2.199	**0.027**	0.833	5.255
Multivariate Cox model radiomics	original_shape_Elongation	–0.056	0.944	0.267	–0.212	0.831	0.559	1.594
	original_shape_MajorAxisLength	0.362	1.437	0.242	1.495	0.134	0.893	2.312
	original_shape_Sphericity	–0.083	0.919	0.246	–0.338	0.735	0.566	1.492
	original_glcm_ClusterTendency	0.266	1.305	0.204	1.301	0.193	0.873	1.951
	original_gldm_LowGrayLevelEmphasis	–0.018	0.982	0.311	–0.058	0.953	0.533	1.808
	wavelet.LLH_firstorder_Mean	–0.568	0.566	0.312	–1.822	0.068	0.307	1.043
	wavelet.LLH_glrlm_RunEntropy	–0.833	0.434	0.318	–2.613	**0.008**	0.232	0.811
	wavelet.HHH_glszm_SmallAreaEmphasis	0.441	1.554	0.337	1.308	0.190	0.802	3.010

	square_glszm_SizeZoneNonUniformityNormalized	–0.550	0.576	0.272	–2.018	**0.043**	0.337	0.984
	exponential_firstorder_Minimum	–0.424	0.654	0.229	–1.846	0.064	0.417	1.026
Multivariate Cox model combined	Grade	1.225	3.405	0.535	2.289	**0.022**	1.193	9.720
	Smoker	–0.020	0.979	0.278	–0.074	0.940	0.567	1.690
	CIS	0.738	2.093	0.469	1.573	0.115	0.833	5.255
	original_shape_Elongation	0.026	1.026	0.292	0.090	0.928	0.578	1.823
	original_shape_MajorAxisLength	0.391	1.479	0.240	1.626	0.103	0.922	2.372
	original_shape_Sphericity	0.060	1.062	0.281	0.214	0.830	0.611	1.845
	original_glcm_ClusterTendency	0.403	1.497	0.214	1.882	0.059	0.983	2.279
	original_gldm_LowGrayLevelEmphasis	–0.166	0.846	0.318	–0.522	0.601	0.453	1.581
	wavelet.LLH_firstorder_Mean	–0.490	0.612	0.312	–1.572	0.115	0.332	1.128
	wavelet.LLH_glrlm_RunEntropy	–1.040	0.353	0.346	–3.001	**0.002**	0.179	0.697
	wavelet.HHH_glszm_SmallAreaEmphasis	0.266	1.305	0.348	0.765	0.443	0.659	2.582
	square_glszm_SizeZoneNonUniformityNormalized	–0.610	0.543	0.325	–1.874	0.060	0.287	1.028
	exponential_firstorder_Minimum	–0.430	0.649	0.239	–1.802	0.0714	0.406	1.0384

Integrated analysis of clinical and radiomics variables for enhanced predictive models

By applying Cox proportional hazards regression models and LASSO regression, we identified significant predictors within clinical and radiomics datasets and assessed the combined predictive power of these variables (Figure S8A). The predictive models, constructed using selected features identified through LASSO regression, offered insights into the predictive power of various clinical and radiomics factors in Cox proportional hazards frameworks. These models were developed separately for clinical (Figure S5A), radiomics (Figure S8B), and combined (Figure S8C) datasets, providing a comprehensive analyses.

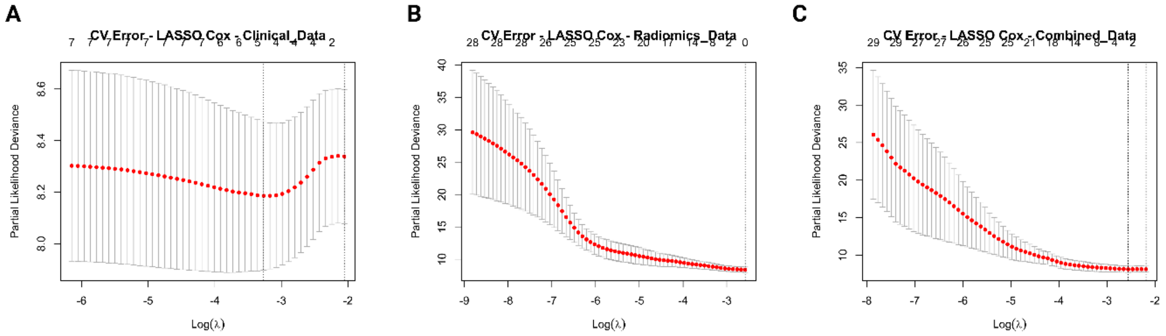

Figure S8. Model selection in multivariate Cox proportional hazards analysis: cross-validation error plots for (A) clinical, (B) radiomics, and (C) combined datasets using LASSO regression in predictive modelling and Cox proportional hazards framework.

The clinical model provided predictors such as grade, smoking, and CIS. Smoker (HR=0.597, p=0.029) and CIS (HR=2.393, p=0.027) showed statistical significance ($p<0.05$), suggesting that being a smoker was associated with a decreased risk of the event by approximately 40%, while the presence of CIS more than doubled the risk. In the radiomics model, features like original_shape_Elongation, original_glcm_ClusterTendency, and wavelet.LLH_glrlm_RunEntropy were examined. Among these is wavelet.LLH_glrlm_RunEntropy had a significant inverse relationship to the risk (HR=0.497, p=0.023), indicating its potential as a predictive biomarker.

In the combined model, original_glcm_ClusterTendency demonstrated a significant positive association with the risk (HR=1.499, p=0.011), highlighting its prognostic significance. Other variables, although not significant, contributed to the depth of the analysis, and revealed complex interactions between clinical and radiomics factors. Furthermore, the models displayed adequate discriminative ability, as evidenced by C-indices of 0.717, 0.750, and 0.730

for the clinical, radiomics, and combined models. The validity of these models is further reinforced by the results from likelihood ratio, Wald, and log-rank tests, which confirmed the fit of the models, and the significance of the variables included.

Evaluation of model fit, complexity, and assumptions in prognostic modeling

Our analysis showed that the clinical model had an AIC of 249.4418 and a BIC of 257.4321, reflecting a moderate fit, whereas the radiomics model demonstrated a better AIC of 235.9877 but a higher BIC of 262.6221, indicating greater complexity. Integrating clinical and radiomics data, the combined model achieved the lowest AIC at 232.4837, suggesting the best fit among the three models. However, it also displayed the highest complexity with a BIC of 267.1084 (Figure S9A). Additionally, proportional hazards testing was conducted to verify the Cox models' assumption of constant HRs, revealing significant violations for specific variables, such as grade and wavelet.HHH_glszm_SmallAreaEmphasis across the models (Figure S9B).

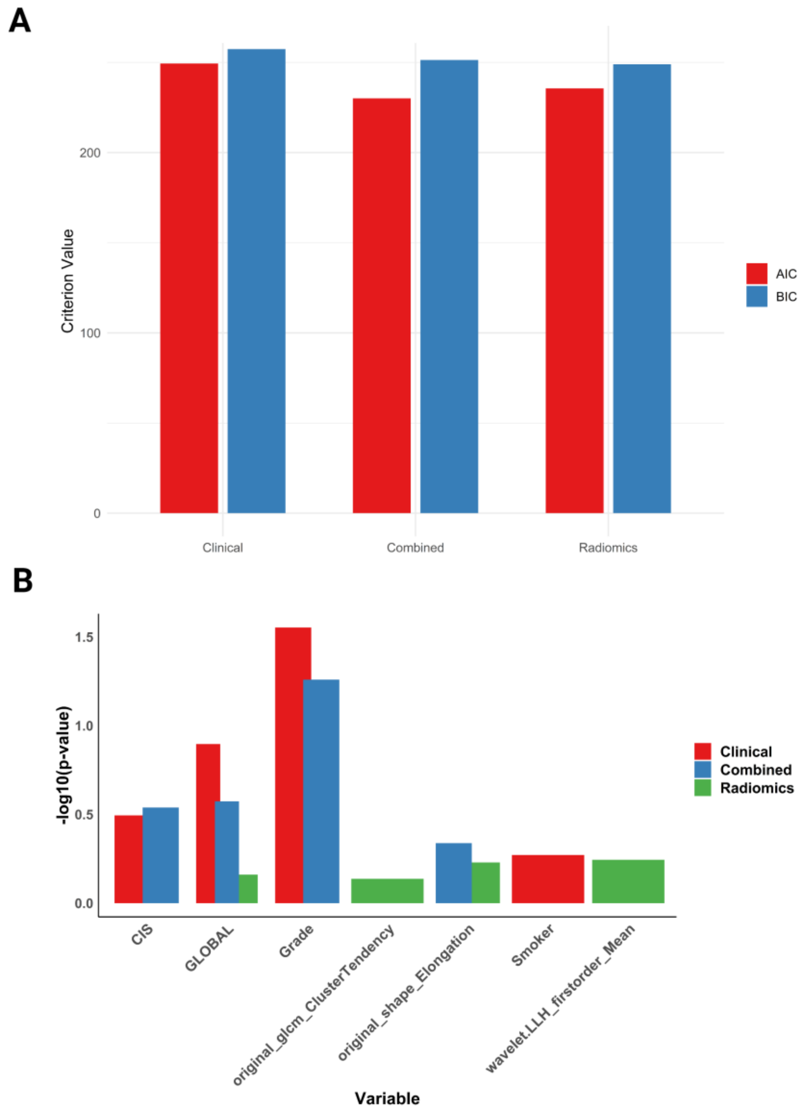

Figure S9. (A) The AIC (red) and BIC (blue) values for clinical, combined, and radiomics Cox proportional hazards models. Lower values suggest a better model fit and *vice versa*. (B) The negative log-transformed p-values (–log10(p-value)) from the proportional hazards test for significant variables in clinical (red), radiomics (green), and combined (blue) models.

Predictive model evaluation: survival analysis with KM plots and risk stratification

The KM method and risk stratification were used to assess survival outcomes across various risk groups in clinical, radiomics, and combined models, while the risk scores were computed for each dataset utilising fitted Cox proportional hazards models categorising individuals into low-, medium-, and high-risk groups (Figure S10). Significant differences in survival probabilities were evident for the clinical model, with high-risk patients exhibiting the lowest survival over time (p=0.00072; Figure S11A). The radiomics model also differentiated risk levels markedly, with its high-risk group showing a considerable decline in survival probability (p=0.00082; Figure S11B).

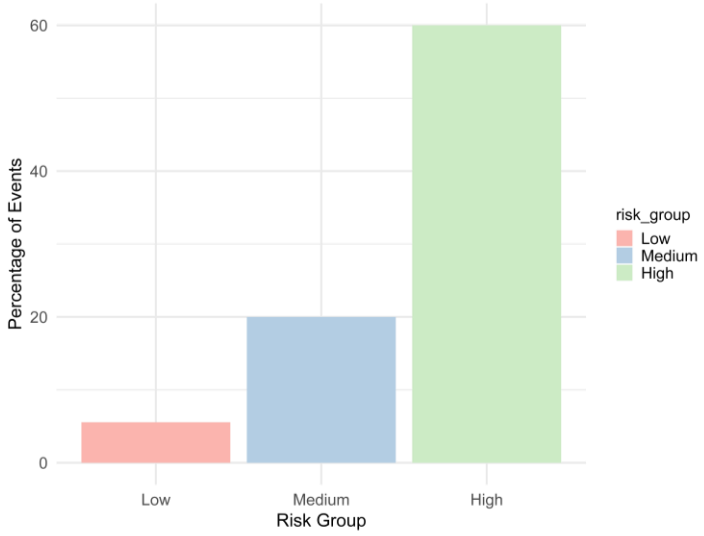

Figure S10. Bar diagram showing stratification of risk groups derived from KM survival analysis in a study examining different risk levels in clinical, radiomics, and combined models. Individuals were classified into three tiers—low (red), medium (blue), and high (green) risk—based on the scores from the fitted Cox proportional hazards models.

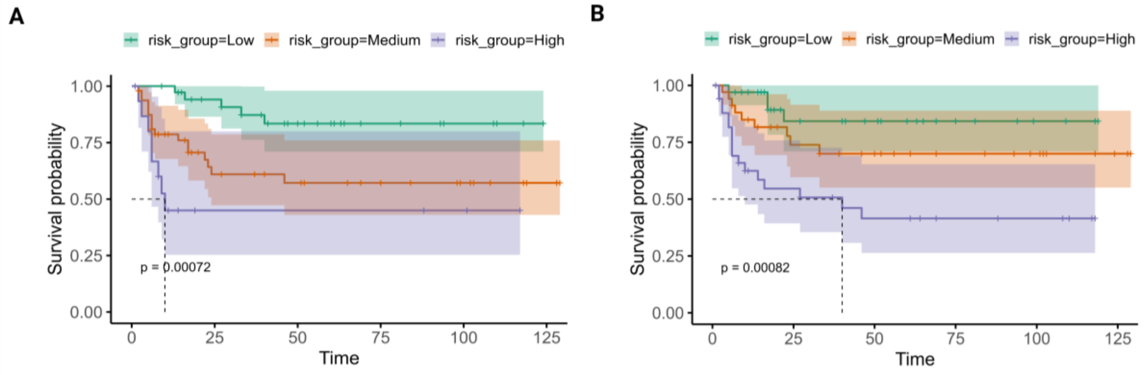

Figure S11. The survival probabilities over time for high-risk (blue) and low-risk (green) groups across (A) clinical, and (B) radiomics models spanning 125 months. The median survival trajectory (red line) for a group of patients, with "+" symbols marking censored data points due to patient loss to follow-up or termination of the study before an event occurrence, is shown. The separation between high- and low-risk curves illustrated the discriminative power of the models, while the shaded region around the median survival indicated 95% CIs.

Visualising temporal evolution of discriminatory power: time-dependent AUC analysis.

The AUC was a pivotal metric for gauging model efficacy in the evaluation of predictive models for forecasting patient outcomes across multiple timepoints. Analysis revealed the clinical model's moderate predictive prowess, with an AUC of 0.711 (60 months)–0.802 (12 months), indicating its reasonable discriminative capability, particularly over short to medium terms (Figure S12A). Conversely, the radiomics model, leveraging sophisticated imaging features, performed better with an AUC of 0.876 at 12 months, showcasing its ability to differentiate patient outcomes, especially in the first year (Figure S12B).

The clinical model began with a high AUC that decreased and then plateaued, indicating an initial (vital) predictive accuracy that stabilised over time. The radiomics model showed a consistent AUC throughout the evaluated periods, suggesting steady predictive performance. On the contrary, the combined model demonstrated an AUC that decreased slightly before stabilising, reflecting an initial reduction in predictive accuracy.

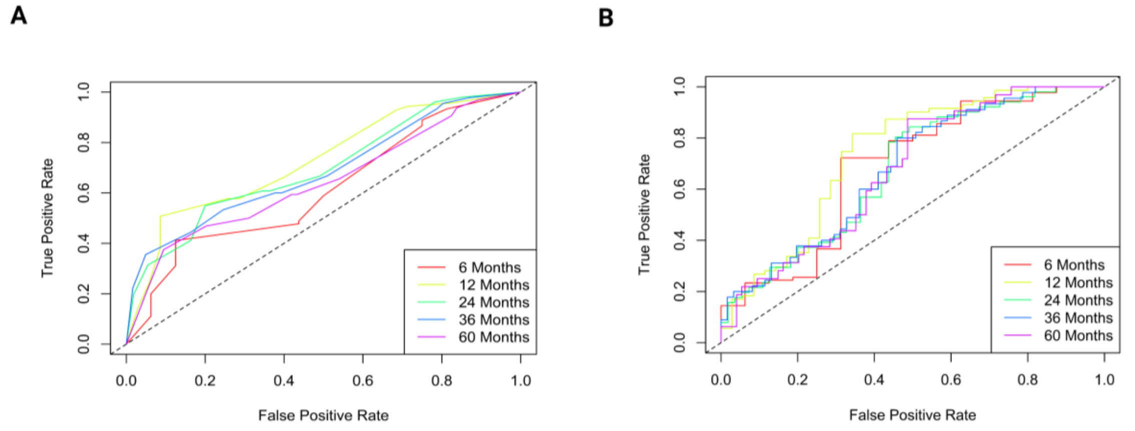

Figure S12. The ROC curves of (A) clinical, and (B) radiomics models with data plotted over 6-, 12-, 24-, 36-, and 60-month periods.
